# The RNAPII-CTD Maintains Genome Integrity through Inhibition of Retrotransposon Gene Expression and Transposition

**DOI:** 10.1371/journal.pgen.1005608

**Published:** 2015-10-23

**Authors:** Maria J. Aristizabal, Gian Luca Negri, Michael S. Kobor

**Affiliations:** 1 Centre for Molecular Medicine and Therapeutics, Child and Family Research Institute, Department of Medical Genetics, University of British Columbia, Vancouver, British Columbia, Canada; 2 Department of Molecular Oncology, BC Cancer Research Center, University of British Columbia, Vancouver, British Columbia, Canada; University of Oxford, UNITED KINGDOM

## Abstract

RNA polymerase II (RNAPII) contains a unique C-terminal domain that is composed of heptapeptide repeats and which plays important regulatory roles during gene expression. RNAPII is responsible for the transcription of most protein-coding genes, a subset of non-coding genes, and retrotransposons. Retrotransposon transcription is the first step in their multiplication cycle, given that the RNA intermediate is required for the synthesis of cDNA, the material that is ultimately incorporated into a new genomic location. Retrotransposition can have grave consequences to genome integrity, as integration events can change the gene expression landscape or lead to alteration or loss of genetic information. Given that RNAPII transcribes retrotransposons, we sought to investigate if the RNAPII-CTD played a role in the regulation of retrotransposon gene expression. Importantly, we found that the RNAPII-CTD functioned to maintaining genome integrity through inhibition of retrotransposon gene expression, as reducing CTD length significantly increased expression and transposition rates of Ty1 elements. Mechanistically, the increased Ty1 mRNA levels in the *rpb1-CTD11* mutant were partly due to Cdk8-dependent alterations to the RNAPII-CTD phosphorylation status. In addition, Cdk8 alone contributed to Ty1 gene expression regulation by altering the occupancy of the gene-specific transcription factor Ste12. Loss of *STE12* and *TEC1* suppressed growth phenotypes of the RNAPII-CTD truncation mutant. Collectively, our results implicate Ste12 and Tec1 as general and important contributors to the Cdk8, RNAPII-CTD regulatory circuitry as it relates to the maintenance of genome integrity.

## Author Summary

RNA Polymerase II (RNAPII) is the enzyme responsible for the transcription of all protein-coding genes, many non-coding genes and retrotransposons. RNAPII has a unique C-terminal domain (CTD) that is composed of repeats of a seven amino acid sequence and is conserved across species. The CTD functions as a recruiting platform for regulatory and RNA processing factors, making the CTD a master orchestrator of transcription. Mutants containing reduced CTD length show defects in steady state transcription during growth in normal conditions, as well as defects in induced gene expression. However, how CTD length affects retrotransposon gene expression remains to be investigated. Here, we uncovered a direct role for CTD length in limiting retrotransposon gene expression and mobility, revealing a new role for the RNAPII-CTD in maintaining genome integrity.

## Introduction

RNA polymerase II (RNAPII) is the enzyme responsible for the transcription of a diverse set of genomic loci, including most protein coding genes, many non-coding genes, and retrotransposons. Rpb1, the largest subunit of RNAPII, contains a unique C-terminal domain (CTD) that is composed of heptapeptide repeats (Y_1_ S_2_ P_3_ T_4_ S_5_ P_6_ S_7_), the number of which increases with genomic complexity [1,2]. The CTD plays key roles in the regulation and coordination of co-transcriptional processes *in vivo*, a function linked to its ability to be differentially phosphorylated during the transcription cycle [3–5]. Generally, the RNAPII-CTD is phosphorylated at S_5_ and S_7_ residues at the 5’ end of genes, where RNAPII-CTD S_5_ phosphorylation functions in the release of RNAPII from promoter elements [6–8]. Conversely, the RNAPII-CTD is phosphorylated at S_2_ residues towards the 3’ end of genes, and this modification plays important roles in coordinating the sequential recruitment of elongation and termination factors [9,10]. In the budding yeast, *Saccharomyces cerevisiae*, deletion of the entire CTD is lethal, while strains carrying shortened versions are viable but display a range of conditional phenotypes, including reduced growth when exposed to high or low temperatures, inositol-deplete conditions, or to the genotoxic agents formamide and hydroxyurea. [11–15]. CTD truncation mutants also lead to alterations in steady state transcription when grown under normal conditions, as evidenced by decreased or increased mRNA and RNAPII levels at a subset of genes, the later primarily regulated by the transcription factor Rpn4 [12]. In addition, CTD truncation mutants have induction defects at the *INO1* and *GAL4* genes [14]. Interestingly, loss of the gene encoding for the Cdk8 kinase subunit of the Mediator complex restores many CTD length-dependent growth and gene expression alterations, establishing it as an important contributor to the RNAPII-CTD regulatory circuitry [11,12].

Retrotransposons constitute a major group of genetic elements transcribed by RNAPII, comprising over 3% of the genome and accounting for 5–10% of the total mRNA in haploid yeast [16,17]. In *S*. *cerevisiae*, retrotransposons are flanked by long terminal repeats (LTR), which contain promoter and termination sequences required for their transcription [18]. Retrotransposons contain a *gag* gene, which encodes a structural coat protein, and a *pol* gene, which encodes a polypeptide that is processed into the enzymes reverse transcriptase, protease and integrase. A crucial step in the replication cycle of retrotransposons is the production of a RNA intermediate by RNAPII [19]. Retrotransposon RNA is required for the synthesis of its proteins and as a template for the synthesis of cDNA, the material that becomes integrated into a new genomic location. Newly integrated copies of retrotransposon cDNA can be transcribed by RNAPII thus giving rise to a new replication cycle.

Transposition events can have grave consequences for genome structure and function, making retrotransposons important sources of genome instability [20]. Specifically, integration within host genes, although rare, can result in disruption of genetic information, while insertion within a transcription regulatory region can alter the expression of adjacent genes [21,22]. To restrict genome instability caused by transposition, all stages in the retrotransposon’s multiplication cycle are kept under tight control by the host cell. For example, in diploid yeast Ty1 gene expression is limited by the a1-alpha2 mating repressor pair [23]. Nonetheless, cellular stress resulting from genetic alterations, exposure to DNA damage conditions or adenine starvation, can result in transcriptional activation of retrotransposons leading to subsequent challenges to genomic integrity through increased Ty1 mobility [24–26]. To drive their expression, retrotransposons exploit several host transcriptional activators including Ste12 and Tec1, both of which drive basal Ty1 transcription in haploid yeast. Supporting a functional role, loss of *TEC1* significantly reduces both steady state Ty1 mRNA levels and transposition rates, while its overexpression results in increased rates of Ty1 transposition [27–34].

The *S*. *cerevisiae* genome contains multiple retrotransposons, belonging to five families called Ty1 to Ty5 [17,35]. These families are closely related but differ in the order and sequence composition of their encoded genes, with Ty1 and Ty2 elements being further divided into subfamilies. Furthermore, only members of Ty1, Ty2 and Ty3 families are capable of transposition, whereas Ty4 and Ty5 elements are likely inactive due to the accumulation of deleterious mutations [36]. In addition, the yeast genome also contains LTR fragments and lone LTRs, known as delta, sigma, tau, and omega elements [17]. These are LTR sequences remaining in the genome following homologous recombination between the almost identical LTRs flanking retrotransposon, and as such their sequence and location provide a record of previous retrotransposon integration events.

Building on previous work from our laboratory, which investigated the role of the RNAPII-CTD in the expression of most protein coding genes, we focused here on examining its contribution to retrotransposon biology [12]. We found that the RNAPII-CTD had an important role in regulating RNAPII and mRNA levels at Ty1 retrotransposons. Shortening RNAPII-CTD length increased Ty1 mRNA levels and transposition rates, suggesting that the structural integrity of the RNAPII-CTD was important for limiting genome instability caused by transposition events. Several lines of evidence suggested that early events in transcription were important in mediating the enhanced expression of retrotransposons in cells with a shortened RNAPII-CTD. We recapitulated the effect using promoter-based reporter assays, and showed that the transcription factors Ste12 and Tec1, and the mediator subunit Cdk8 were required for the increased Ty1 mRNA levels in the RNAPII-CTD truncation mutant. Cdk8 contributed to retrotransposon gene expression by altering the levels of RNAPII-CTD S_5_ phosphorylation, Tec1 and Ste12 at Ty1 promoters. Lastly, suggesting a broader role for these factors in RNAPII CTD function, we found that loss of *STE12* or *TEC1* suppressed RNAPII-CTD truncation mutant growth phenotypes, the latter likely in conjunction with *CDK8*.

## Results

### Truncation of the RNAPII-CTD increased RNAPII occupancy at a subset of retrotransposons

**Fig 1:**
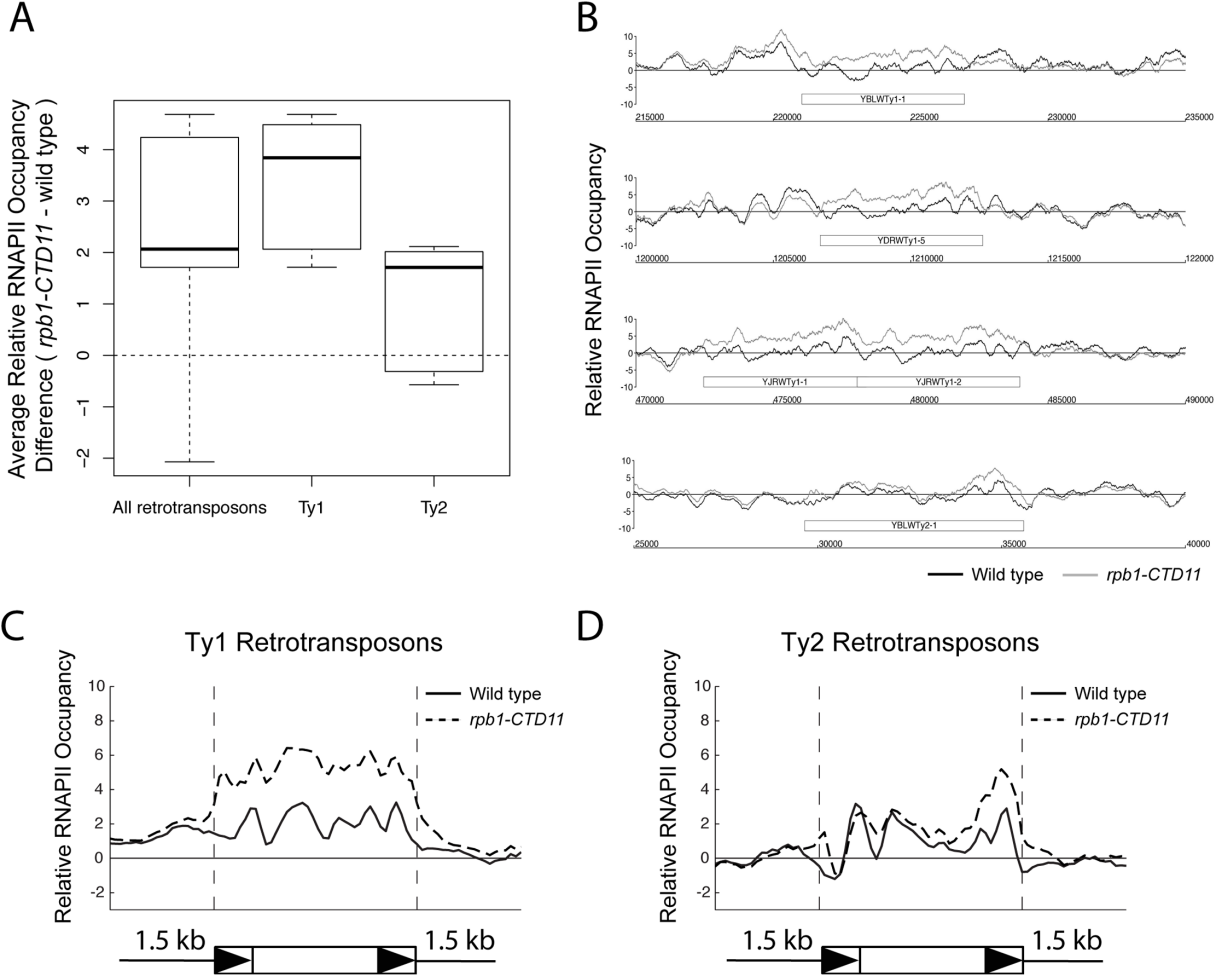
Genome-wide occupancy profiles of RNAPII suggested a role for the RNAPII-CTD in retrotransposon regulation. (A) Box plot showing differences in average MAT RNAPII (Rpb3) occupancy scores between the wild type and the *rpb1-CTD11* mutant at all, Ty1, or Ty2 retrotransposons. (B) Chromosome plots of relative RNAPII occupancy at representative retrotransposons. Increased RNAPII levels were observed in the *rpb1-CTD11* mutant compared to wild type. Labeled boxes indicate the retrotransposon. (C) Average gene profile of RNAPII occupancy at Ty1 retrotransposons showed increased levels along the length of the feature. Below, schematic of an average retrotransposon. Black triangles indicate the LTRs. (D) Average gene profile of RNAPII occupancy at Ty2 retrotransposons revealed increased levels towards the 3’ end of the feature.

Our previous characterization of genes whose expression is dependent on CTD length focused on protein coding genes [12]. To test whether the RNAPII-CTD had a role in the regulation of retrotransposons, we determined whether truncation of the RNAPII-CTD led to alterations in RNAPII (Rpb3 subunit) occupancy at retrotransposons using chromatin immunoprecipitation followed by hybridization to high-density microarrays (ChIP-on-chip). Grouping all 50 retrotransposon elements present in the *S*. *cerevisiae* genome revealed significantly increased RNAPII occupancy levels in the *rpb1-CTD11* mutant, which contained only 11 heptapeptide repeats, compared to wild type (Fig 1A) (Table 1). We then focused on retrotransposon families rather than individual retrotransposons, as the high degree of sequence similarity amongst retrotransposon family members limited our ability to uniquely identify single elements in the ChIP-on-chip platform. Overall, the Ty1 (31 elements) and Ty2 (13 elements) family of retrotransposons had significant CTD length-dependent increases in RNAPII levels, although the effect at Ty1 elements was more pronounced than at Ty2 elements. For Ty1 and Ty2 elements, representative examples of individual retrotransposons further illustrated the differences apparent from the average occupancy profiles (Fig 1B). In addition, average gene profiles revealed that at Ty1 elements truncation of the RNAPII-CTD resulted in elevated RNAPII levels along the length of the entire element, while at Ty2 elements the increased RNAPII levels occurred primarily at their 3’ ends, suggesting different regulatory roles for the RNAPII-CTD in Ty1 and Ty2 biology (Fig 1C and 1D). The Ty3, Ty4 and Ty5 family of retrotransposons (having 2, 3 and 1 element respectively) were not investigated further because the limited number of members in each family prevented meaningful statistical analysis. However, relative RNAPII occupancy profiles at individual retrotransposons revealed that truncation of the RNAPII-CTD resulted in elevated RNAPII levels at Ty3 elements, while no effects were observed at Ty4 and Ty5 elements (S1 Fig).

**Table 1:** Paired t-test p values comparing RNAPII levels in wild type vs *rpb1-CTD11* at Ty1 and Ty1 retrotransposons and derived-LTRs.

Element	One tailed paired t-test p value comparing RNAPII levels in wild type vs *rpb1-CTD11*
All retrotransposons	6.42e-12
Ty1	1.8e-15
Ty2	0.00424
All lone LTRs	1.85e-16
Ty1-derived LTRs	1.34e-11
Ty2-derived LTRs	5.84e-05

To test whether the effect of truncating the RNAPII-CTD was specific to intact retrotransposons, we determined RNAPII occupancy at lone LTRs and found significantly increased levels in the *rpb1-CTD11* mutant compared to the wild type (S2 Fig) (Table 1). Focusing exclusively on delta elements, which are derived from Ty1 and Ty2 elements, revealed that these had significantly increased RNAPII levels in the *rpb1-CTD11* mutant when compared to wild type, consistent with our findings at intact retrotransposons (S2 Fig) (Table 1).

### Truncation of the RNAPII-CTD resulted in altered occupancy of transcription-associated factors at Ty1 and Ty2 retrotransposons

**Fig 2:**
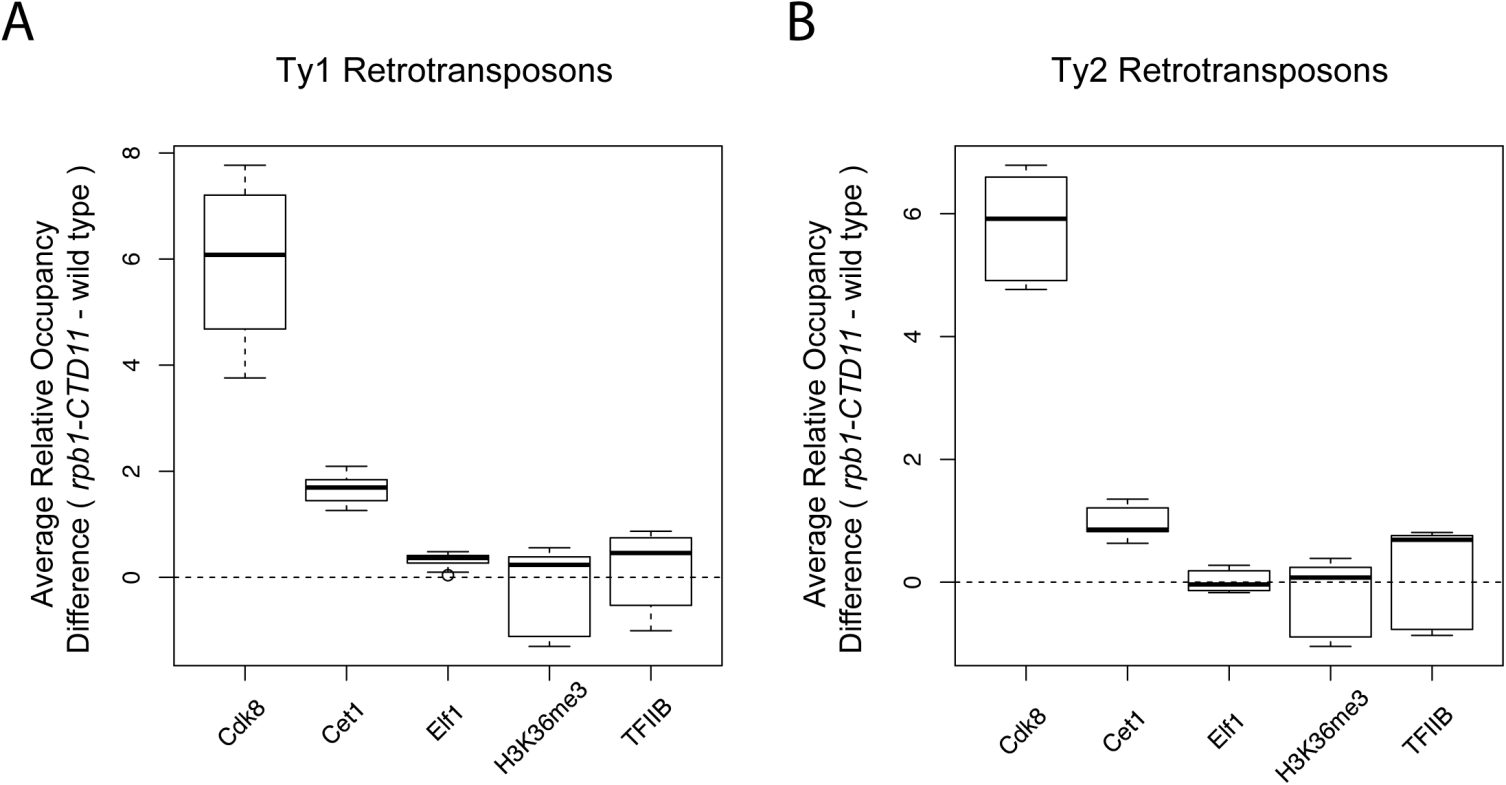
Truncation of the RNAPII-CTD resulted in altered association of a subset of transcription related factors at retrotransposons. Comparison of MAT average occupancy scores at Ty1 (A) or Ty2 (B) retrotransposons for the Mediator subunit, Cdk8, the mRNA capping enzyme, Cet1, the elongation factor, Elf1, the transcription elongation-associated chromatin mark, H3K36me3, and the general transcription factor, TFIIB, under wild type and *rpb1-CTD11* conditions.

To mechanistically understand the effect of truncating the RNAPII-CTD at retrotransposons, we next determined if the increased binding coincided with changes in the occupancy of transcription- or chromatin-related factors at these loci. As such, we took advantage of our previously generated genome-wide occupancy maps of the general transcription factor TFIIB, the Mediator subunit Cdk8, the mRNA capping enzyme Cet1, the elongation factor Elf1, and the transcription elongation-associated chromatin mark H3K36me3 in wild type and the *rpb1-CTD11* mutant [12]. Truncation of the RNAPII-CTD resulted in significantly increased Cdk8, Cet1 and Elf1 occupancy at Ty1 retrotransposons, albeit with clearly different magnitudes (Table 2) (Fig 2A). In contrast, truncating the RNAPII-CTD had no significant effect on the occupancy of TFIIB and H3K36me3 at Ty1 retrotransposons. At Ty2 elements Cdk8 and Cet1 occupancy also showed significantly increased levels in the *rbp1-CTD11* mutant, and no changes were observed for TFIIB and H3K36me3 occupancy (Fig 2B). In contrast to the effect of truncating the RNAPII-CTD at Ty1 elements, Ty2 elements did not show any significant changes in Elf1 occupancy.

**Table 2:** Paired t-test p values comparing the levels of transcription or chromatin-related factors in wild type vs *rpb1-CTD11* at all retrotransposons.

Element	Factor	One tail paired t-test p value comparing wild type vs *rpb1-CTD11*
Ty1	**Cdk8**	**3.59e-21**
	**Cet1**	**1.42e-27**
	**Elf1**	**4.86e-17**
	H3K36me3	0.0199
	TFIIB	0.473
Ty2	**Cdk8**	**1.07e-11**
	**Cet1**	**3.93e-9**
	Elf1	0.672
	H3K36me3	0.205
	TFIIB	0.505

### Structural integrity of the RNAPII-CTD was important for maintaining normal Ty1 mRNA levels and transposition rates

**Fig 3:**
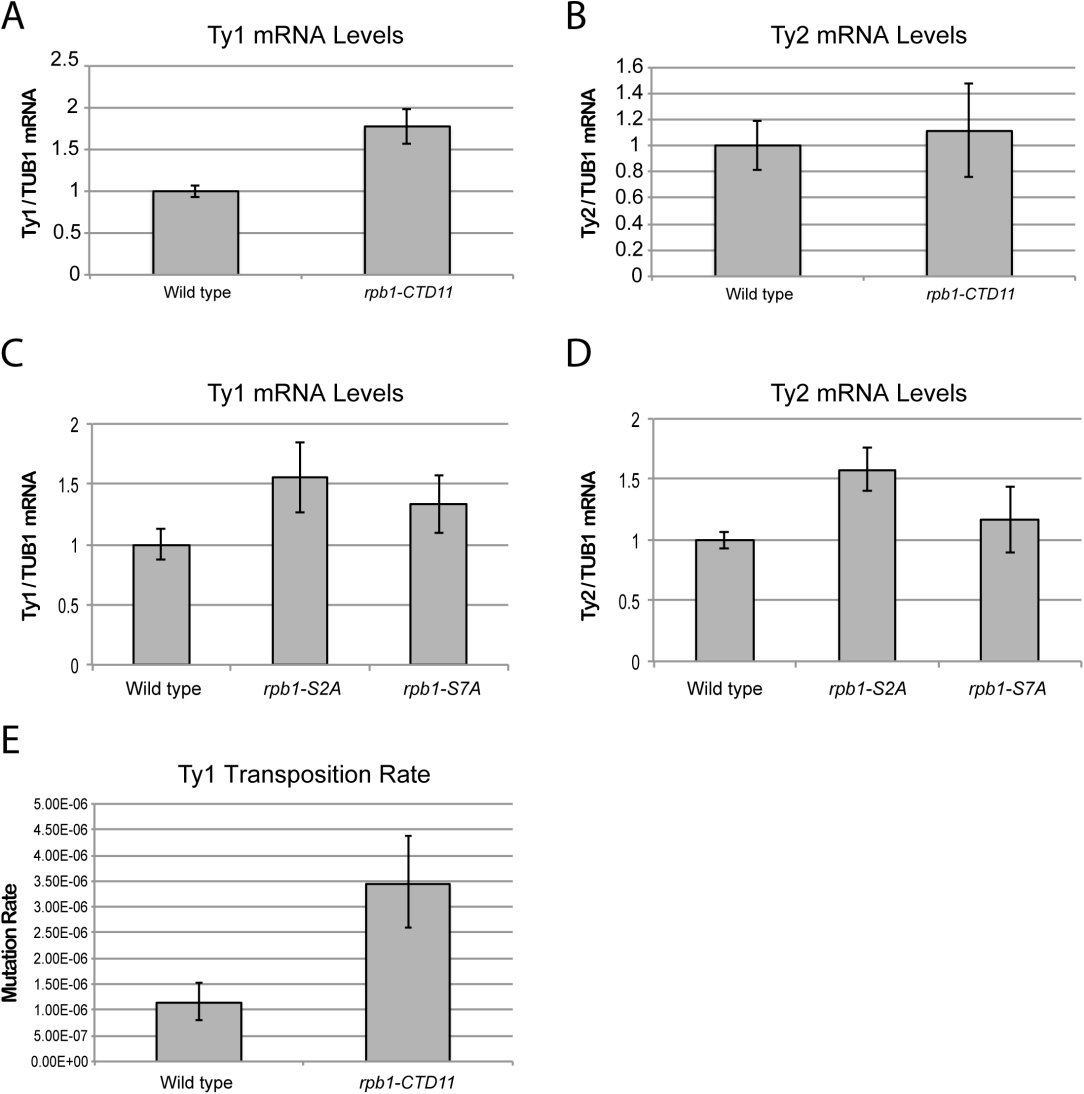
Structural integrity of the RNAPII-CTD was important for normal retrotransposon mRNA levels and transposition rates. (A) Ty1 mRNA levels were significantly increased in the *rpb1-CTD11* mutant compared to wild type. (B) RT-qPCR analysis of Ty2 mRNA levels in the *rpb1-CTD11* mutant compared to wild type. RNAPII-CTD S_2_ phosphorylation was important for maintaining normal Ty1 (C) and Ty2 mRNA levels (D). (E) Transposition rates for Ty1 were increased in the *rpb1-CTD11* mutant compared to wild type. Error bars represent 95% confidence intervals. Transposition rates and confidence intervals were calculated using the Fluctuation AnaLysis CalculatOR (FALCOR) web tool.

Given the increased RNAPII levels at Ty1 and Ty2 retrotransposons in the *rpb1-CTD11* mutant, we hypothesized that concurrent changes in the mRNA levels of these elements would occur. We designed an RT-qPCR based assay to quantitatively measure Ty mRNA levels, focusing on regions that were unique to all members of a single retrotransposon family, and compared the levels to those of a control protein coding gene, *TUB1* whose mRNA levels are not altered upon truncation of the RNAPII-CTD [12]. Mirroring the RNAPII occupancy data, mRNA levels of Ty1 retrotransposons were significantly increased in the *rpb1-CTD11* mutant compared to wild type (Fig 3A). Ty2 retrotransposons had a tendency for modestly increased mRNA levels although this did not reach statistical significance (Fig 3B). The latter was consistent with the weaker effect of truncating the RNAPII-CTD on RNAPII occupancy levels at Ty2 elements. Taking advantage of a set of available mutants lacking S_2_ (*rpb1-S2A*) and S_7_ (*rpb1-S7A*) phosphorylation sites on the RNAPII-CTD [37], we also investigated the importance of CTD phosphorylation on retrotransposon gene expression. Loss of S_7_ phosphorylation had no effect on Ty1 or Ty2 mRNA levels (Fig 3C and 3D). In contrast, the *rpb1-S2A* mutant resulted in significantly increased Ty1 and Ty2 mRNA levels, revealing a broader role for the RNAPII-CTD in retrotransposon gene expression regulation.

Having established that upon truncation of the RNAPII-CTD Ty1 mRNA levels significantly increased, we sought to determine if this had functional consequences on genome stability manifested by increased transposition rates. Using an established Ty1 cDNA-mediated mobility assay in living yeast cells [38,39], we measured transposition rates in wild type and *rpb1-CTD11* mutants. Demonstrating that genomic integrity was indeed compromised upon loss of the RNAPII-CTD repeats, we found that truncation of the RNAPII-CTD resulted in a 3-fold increase in Ty1 transposition rates compared to wild type (Fig 3E).

### Loss of *CDK8* normalized the increased RNAPII and mRNA levels at Ty1 retrotransposons

**Fig 4:**
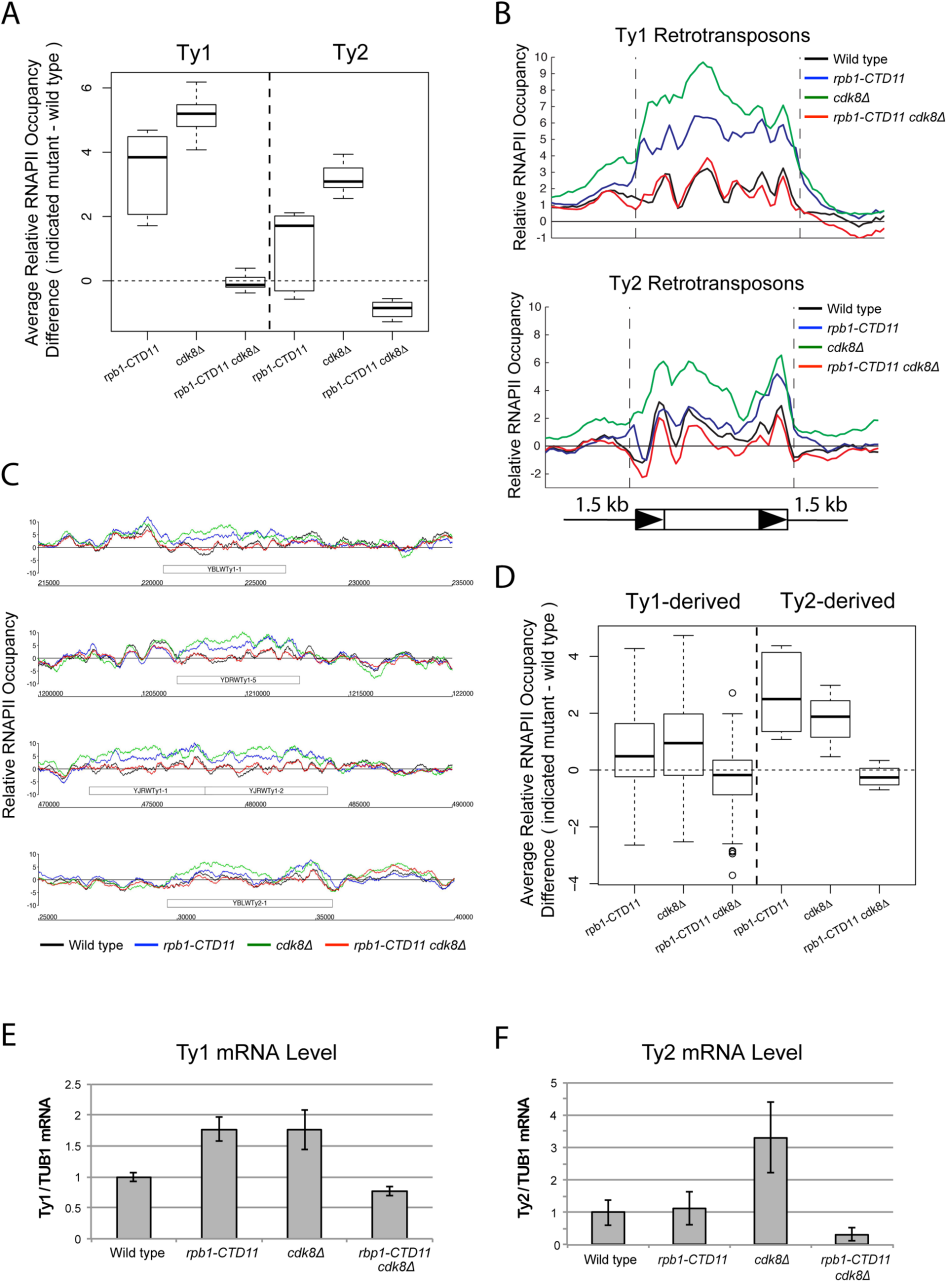
Loss of *CDK8* normalized the elevated RNAPII and mRNA levels at Ty1 and Ty2 retrotransposons. (A) MAT average RNAPII (Rpb3) occupancy scores at retrotransposons revealed elevated levels at Ty1 and Ty2 elements in the single *cdk8Δ* mutant. In the *rpb1-CTD11* background, loss of *CDK8* resulted in normalized RNAPII levels at Ty1 and Ty2 retrotransposons. (B) Average gene profiles of RNAPII occupancy at Ty1 (top) or Ty2 (bottom) retrotransposons showed normalized RNAPII levels upon loss of *CDK8* in the *rpb1-CTD11* mutant. (C) Chromosome plots of RNAPII levels at representative retrotransposons. (D) Loss of *CDK8* normalized the elevated RNAPII levels at Ty1- and Ty2-derived LTRs observed in the *rpb1-CTD11* mutant. (E) RT-qPCR analysis of wild type, *rpb1-CTD11*, *cdk8Δ* and *rpb1-CTD11 cdk8Δ* revealed that loss of *CDK8* significantly normalized the elevated mRNA levels of Ty1 elements in the *rpb1-CTD11* background. (F) Ty2 mRNA levels were significantly elevated in the *cdk8Δ* mutant, an effect that was normalized when combined with an RNAPII-CTD truncation.

Given that loss of *CDK8* can suppress a number of RNAPII-CTD truncation mutant phenotypes [11,12], and Cdk8 occupancy was increased at Ty1 and Ty2 retrotransposons in the *rpb1-CTD11* mutant, we hypothesized that Cdk8 might contribute to the increased RNAPII occupancy and mRNA levels at Ty elements upon truncation of the CTD. Focusing on Ty1 and Ty2 elements, we did find that RNAPII levels were restored to wild type levels in the *rpb1-CTD11 cdk8*Δ double mutant, as evidenced by average occupancy scores and average gene profiles (Fig 4A and 4B) (Table 3). Specifically, average RNAPII binding scores at Ty1 and Ty2 elements in the *rpb1-CTD11 cdk8Δ* double mutant were significantly lower than the scores of the *rpb1-CTD11* mutant and were not statistically different from the scores of wild type cells. Furthermore, RNAPII occupancy patterns at representative individual retrotransposons also showed restoration to wild type levels caused by loss of *CDK8* in the *rpb1-CTD11* mutant (Fig 4C). A similar effect was observed at Ty1- and Ty2-derived LTRs (Fig 4D). Importantly, changes in RNAPII occupancy were mirrored by changes in mRNA levels at Ty1 retrotransposons, as loss of *CDK8* also restored the elevated mRNA levels in the *rpb1-CTD11* mutant to wild type levels (Fig 4E).

**Table 3:** Paired t-test p values comparing the levels of RNAPII at Ty1 and Ty2 retrotransposons and Ty-derived LTRs.

Element	Comparison	One tail paired t-test p value
Ty1	Wild type vs *rpb1-CTD11*	1.8e-15
	Wild type vs *cdk8Δ*	3.68e-32
	Wild type vs *rpb1-CTD11 cdk8Δ*	0.896
	*rpb1-CTD11* vs *rpb1-CTD11 cdk8Δ*	2.01e-17
Ty1-derived LTRs	Wild type vs *rpb1-CTD11*	1.34e-11
	Wild type vs *cdk8Δ*	9.58e-18
	Wild type vs *rpb1-CTD11 cdk8Δ*	1
	*rpb1-CTD11* vs *rpb1-CTD11 cdk8Δ*	3.08e-22
Ty2	Wild type vs *rpb1-CTD11*	0.00424
	Wild type vs *cdk8Δ*	6.24e-12
	Wild type vs *rpb1-CTD11 cdk8Δ*	1
	*rpb1-CTD11* vs *rpb1-CTD11 cdk8Δ*	5.68e-06
Ty2-derived LTRs	Wild type vs *rpb1-CTD11*	5.84e-05
	Wild type vs *cdk8Δ*	4.63e-05
	Wild type vs *rpb1-CTD11 cdk8Δ*	0.946
	*rpb1-CTD11* vs *rpb1-CTD11 cdk8Δ*	2.3e-05

Additional inspection of the RNAPII occupancy profiles revealed that loss of *CDK8* alone resulted in significantly elevated average RNAPII levels at Ty1 and Ty2 retrotransposons when compared to wild type (Fig 4A, 4B and 4C) (Table 3). Most interestingly, the increased RNAPII levels in the *cdk8Δ* mutant were significantly reduced in the *rpb1-CTD11 cdk8Δ* double mutant, demonstrating that the suppression of the elevated RNAPII levels at Ty1 and Ty2 elements between the RNAPII-CTD mutant and *CDK8* deletion was reciprocal. The changes in RNAPII levels coincided with changes in Ty1 and Ty2 mRNA levels, as loss of *CDK8* resulted in significant increased Ty1 and Ty2 mRNA levels, which were normalized upon truncation of the RNAPII-CTD (Fig 4F). Collectively, these data suggested that the specific *CDK8*-dependent phenotypes could be normalized by functional alteration of the RNAPII-CTD, consistent with a reciprocal repressive relationship at Ty1 and Ty2 elements.

### The *rpb1-CTD11* mutant had elevated RNAPII-CTD S_5_ phosphorylation levels at Ty1 retrotransposons which were normalized by loss of *CDK8*

**Fig 5:**
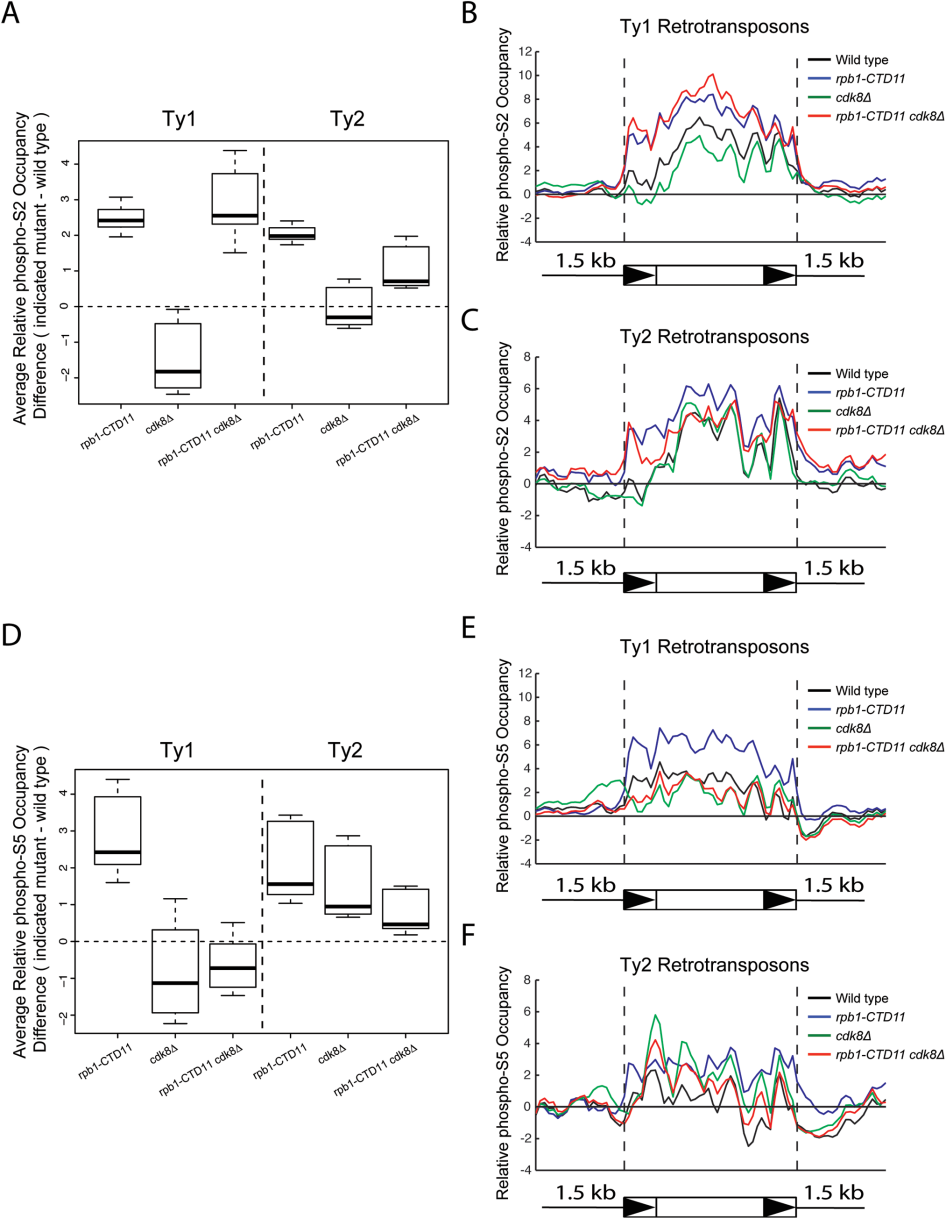
Loss of *CDK8* decreased the elevated RNAPII-CTD S_5_ phosphorylation levels at Ty1 retrotransposons observed in the *rpb1-CTD11* mutant. (A) Box plot showing differences in average RNAPII-CTD S_2_ phosphorylation occupancy scores between the wild type and the *rpb1-CTD11*, *cdk8Δ* and *rpb1-CTD11 cdk8Δ* mutants at Ty1 or Ty2 retrotransposons. Average gene profiles of phospho-S_2_ occupancy at Ty1 (B) or Ty2 (C) retrotransposons. (D) The elevated average RNAPII-CTD S_5_ phosphorylation scores at Ty1 and Ty2 elements in the *rpb1-CTD11* mutant were reduced upon loss of *CDK8*. Average gene profiles of phospho-S_5_ occupancy at Ty1 (E) or Ty2 (F) retrotransposons.

Cdk8 is involved in directly and indirectly regulating RNAPII-CTD phosphorylation levels [7,40,41], thus we determined if CTD phosphorylation levels were associated with the observed changes in retrotransposon gene expression using ChIP-on-chip. Importantly, our profiles of RNAPII-CTD phosphorylation on all genes in the genome were consistent with previously published profiles (S3 Fig)[42–45]. At Ty1 and Ty2 retrotransposons, RNAPII-CTD S_2_ phosphorylation levels increased in the *rpb1-CTD11* mutant compared to wild type and levels remained high in the *rpb1-CTD11 cdk8Δ* double mutant (Fig 5A, 5B and 5C). RNAPII-CTD S_5_ phosphorylation levels were also increased at Ty1 and Ty2 elements in the *rpb1-CTD11* mutant compared to wild type (Fig 5D, 5E and 5F). Most interestingly, RNAPII-CTD S_5_ phosphorylation levels were reduced at Ty1 elements in the *rpb1-CTD11 cdk8Δ* double, an effect that was less prominent at Ty2 elements. Given that the wild type and *rpb1-CTD11*, *cdk8Δ* and *rpb1-CTD11 cdk8Δ* mutants differed substantially in the levels of RNAPII at Ty1 and Ty2 element, we also normalized each RNAPII-CTD S_2_ and S_5_ phosphorylation profile to its corresponding RNAPII profile. Although it is likely that this approach strongly penalizes the signal from strains carrying the *rpb1-CTD11* alleles, given that their potential for acquiring phosphorylation marks is significantly reduced compared to strains with full length CTDs, we observed similar normalizing effects for S_5_ phosphorylation at Ty1 retrotransposons. Specifically, the *rpb1-CTD11* mutant had significantly increased scores compared to wild type and these were normalized upon loss of *CDK8* (S4 Fig).

### Increased Ty1 mRNA alterations were in part due to changes in promoter activity mediated by Ste12 and Tec1

**Fig 6:**
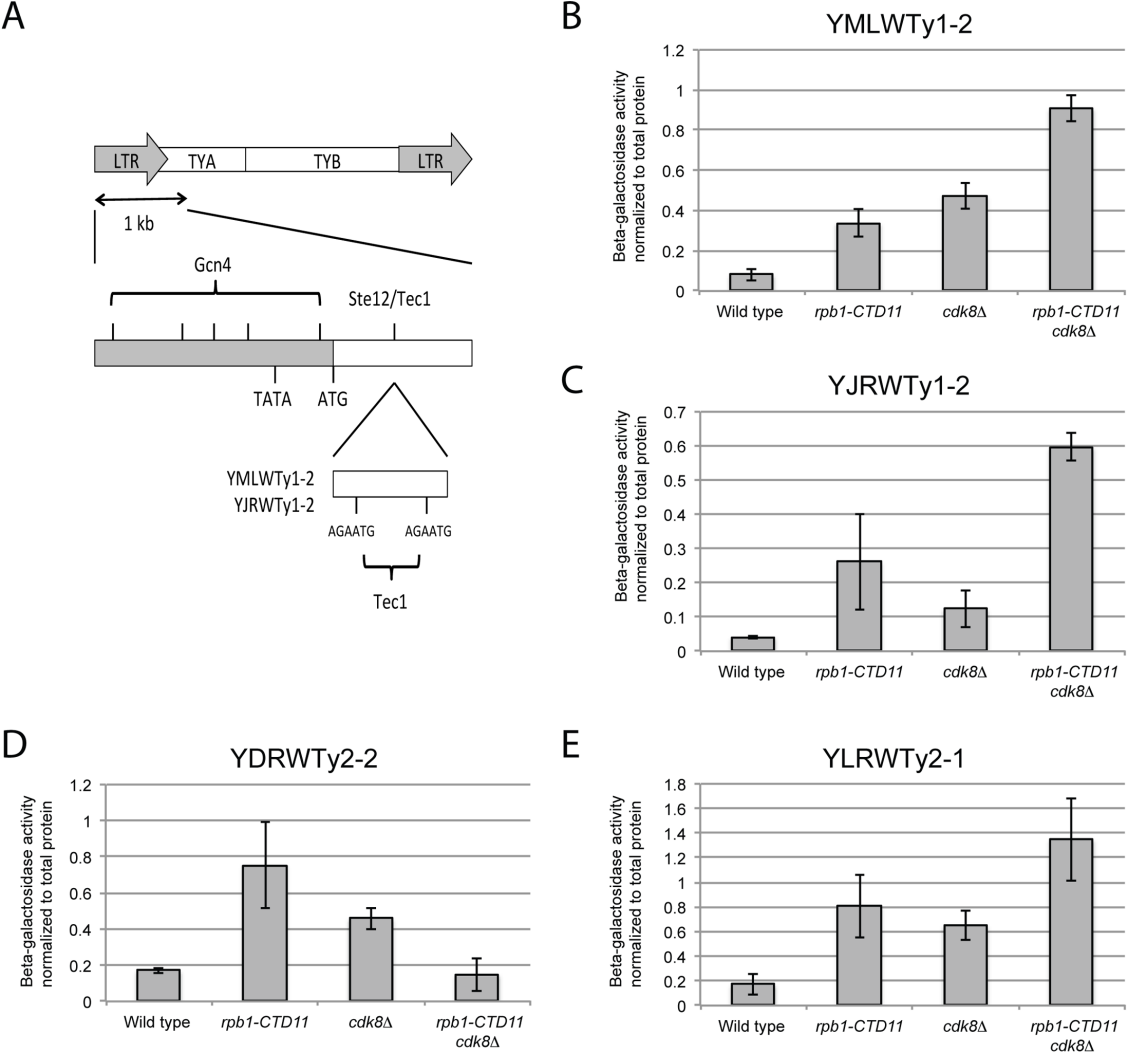
The increased retrotransposon mRNA levels in the *rpb1-CTD11* and *cdk8Δ* mutant were in part due to alterations to promoter activity. (A) Schematic of an average Ty1 promoter with binding sites for Gcn4 and Ste12/Tec1 labeled [48]. TYA and TYB are retrotransposon genes that encode for the coat protein, and reverse transcriptase, protease, integrase, and RNase H respectively. YMLWTy1-2 and YJRWTy1-2 contain two Tec1 binding sites downstream of the ATG start codon, a feature observed on some Ty1 elements. Reporter assays for YMLWTy1-2 (B), YJRWTy1-2 (C), YDRWTy2-2 (D), and YLRWTy2-1 (E) in wild type, *rpb1-CTD11*, *cdk8Δ*, and *rpb1-CTD11 cdk8Δ*.

In *S*. *cerevisiae* the mediator subunit Cdk8 plays major roles in transcription initiation via phosphorylation of transcription factors and the CTD [7,40,46,47]. Thus, finding that loss of *CDK8* normalized the elevated Ty1 mRNA and RNAPII-CTD S_5_ phosphorylation levels of the *rpb1-CTD11* mutant suggested that the regulation likely occurred at the level of transcription initiation. To formally test this possibility, we employed a reporter strategy wherein we inserted more than 1 kb of promoter sequences from representative Ty1 and Ty2 elements into a LacZ reporter plasmid. The representative Ty1 elements selected contained features found in most Ty1 promoters, including putative binding sites for the transcription factors Ste12 and Tec1, which often bind as a heterodimer (Fig 6A) [48,49]. For all representative promoters tested, the reporter assays showed significantly increased β-galactosidase activity in the *rpb1-CTD11* and *cdk8Δ* mutant compared to wild type, suggesting that these promoter sequences alone were sufficient to recapitulate the expression changes of the endogenous retrotransposons observed in these mutants (Fig 6B–6E). However, β-galactosidase activity generally remained high compared to wild type in the *rpb1-CTD11 cdk8Δ* double mutant, suggesting that events beyond those controlled by promoter sequences were involved in normalizing the elevated mRNA and RNAPII levels at retrotransposons.

**Fig 7:**
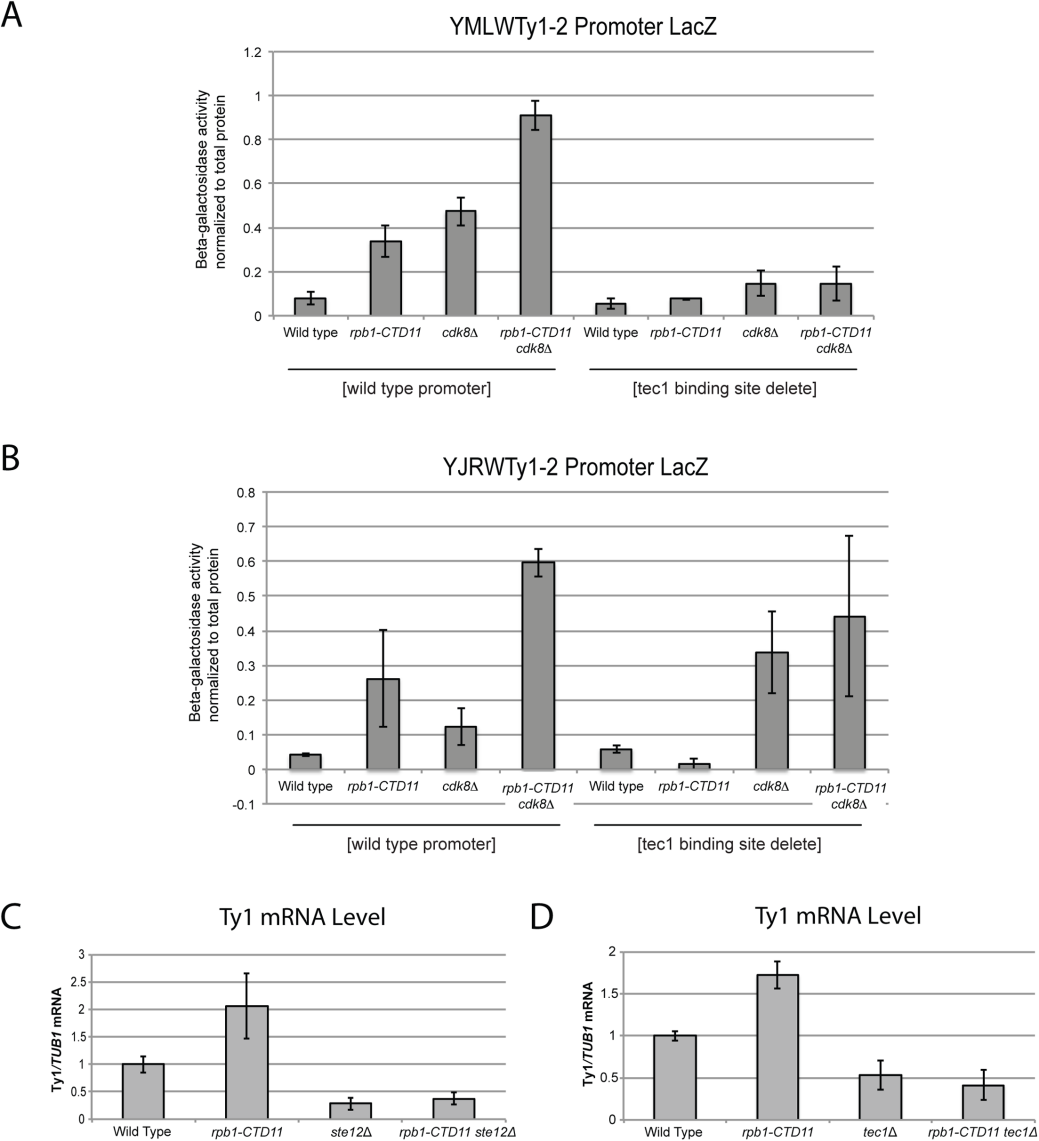
The increased Ty1 gene expression levels observed in the *rpb1-CTD11* mutant were dependent on *TEC1* or *STE12*. (A and B) Reporter assay for YMLWTy1-2 or YJRWTy1-2, with or without deletion of Tec1 binding sites. Tec1 binding sites were required for the increased promoter activity of Ty1 reporter constructs upon truncation of the RNAPII-CTD. (C and D) RT-qPCR analysis of wild type, *rpb1-CTD11*, *ste12Δ* and *rpb1-CTD11 ste12Δ* or wild type, *rpb1-CTD11*, *tec1Δ* and *rpb1-CTD11 tec1Δ* revealed that loss of *STE12* or *TEC1* in the *rpb1-CTD11* background led to Ty1 mRNA levels similar to those observed in the wild type.

Further expanding the mechanistic details of the RNAPII-CTD-dependent regulation of Ty1 elements, we found that removal of the binding sites corresponding to the Tec1 consensus sequence affected expression of our reporter constructs. While, the baseline expression from the YMLWTy1-2 and YJRWTy1-2 reporter constructs was not dependent on intact Tec1 binding sites, their removal was sufficient to abolish the increased transcription of the reporter caused by truncation of the RNAPII-CTD (Fig 7A and 7B). The effect of removing the Tec1 binding sites in the *cdk8Δ* and the *rpb1-CTD11 cdk8Δ* mutant background was more nuanced. For the YMLWTy1-2 reporter construct, removal of the Tec1 binding sites reduced the increased reporter activity. In contrast, for the YJRWTy1-2 reporter construct removal of the Tec1 binding sites did not reduced β-galactosidase activity. In fact, in the *cdk8Δ* mutant removing the Tec1 binding sites exacerbated the transcription defect. In conclusion, although Tec1-dependent regulation was required for the increased Ty1 expression levels observed in the *rpb1-CTD11* mutant, individual Ty1 elements differed in their requirement for Tec1, as revealed when *CDK8* was mutated.

**Fig 8:**
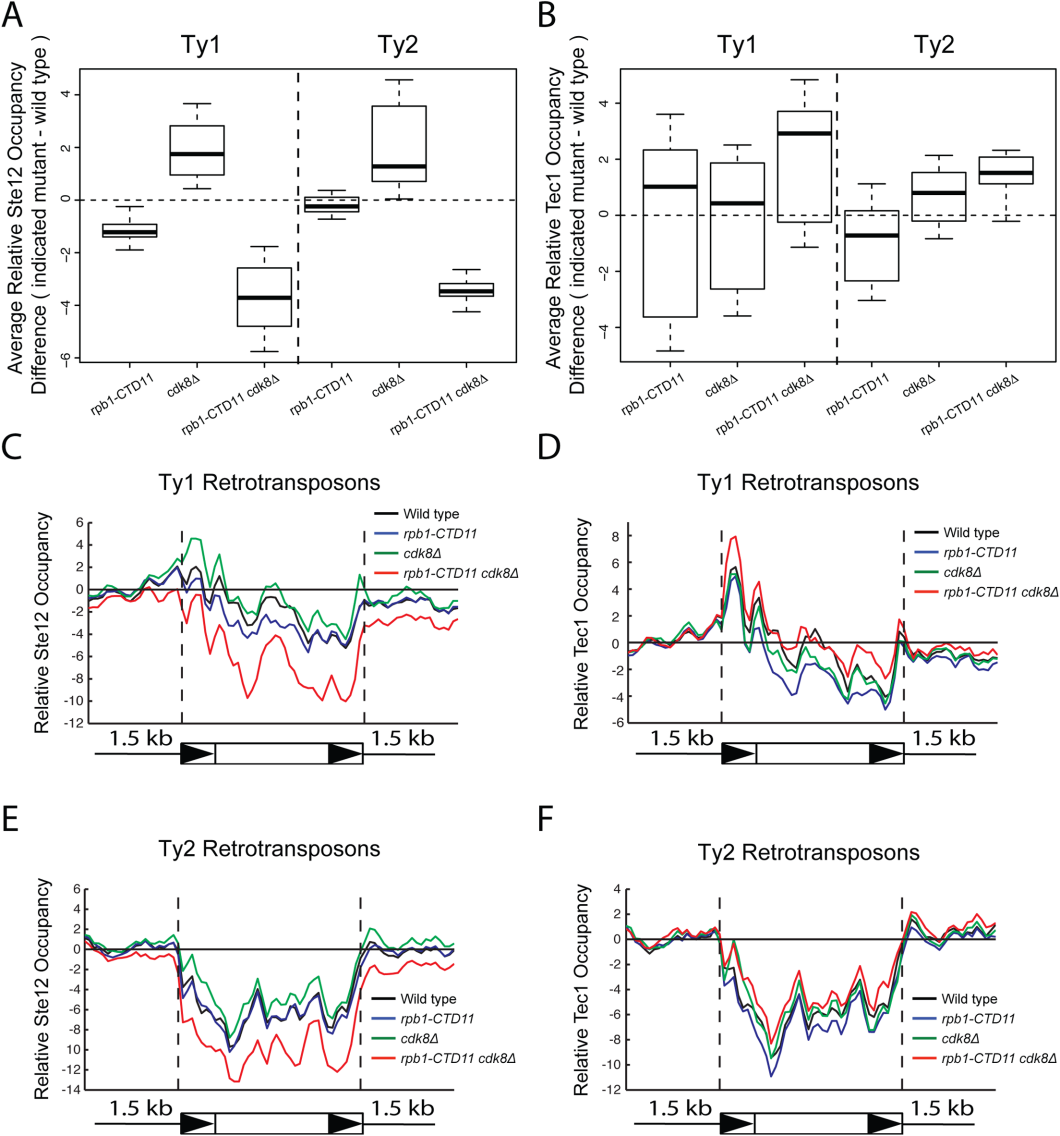
Increased levels of Ste12 at Ty1 and Ty2 promoters in the *cdk8Δ* mutant were normalized by truncation of the RNAPII-CTD. Box plot showing differences in average Ste12 (A) or Tec1 (B) occupancy scores between the wild type and the *rpb1-CTD11*, *cdk8Δ* and *rpb1-CTD11 cdk8Δ* mutant at Ty1 or Ty2 retrotransposons. Average gene profiles of Ste12 (C) or Tec1 (D) occupancy at Ty1 retrotransposons. Average gene profiles of Ste12 (E) or Tec1 (F) occupancy at Ty2 retrotransposons.

Given that the increased expression of Ty1 elements in the RNAPII-CTD truncation mutant resulted in part from Tec1 binding site-dependent alterations in transcription initiation, we next focused on the effect of loss of *TEC1* and its regulatory partner *STE12* on endogenous Ty1 mRNA levels. The connection to both Ste12 and Tec1 as regulatorss of Ty1 expression was particularly intriguing given that both are also directly (Ste12) and indirectly (Tec1) regulated by Cdk8 [50,51]. Consistent with their known roles in Ty1 gene expression [28,33], *ste12Δ* and *tec1Δ* single mutants both had reduced Ty1 mRNA levels compared to wild type (Fig 7C and 7D). More importantly, loss of *TEC1* or *STE12* reduced the elevated Ty1 mRNA levels in the *rpb1-CTD11* mutant. One explanation for the increased Ty1 mRNA levels in the *rpb1-CTD11* mutant could be that the protein levels of Ste12 or Tec1 were increased in the *rpb1-CTD11* mutant, similar to what we observed for Rpn4 [12]. To this end, we observed no significant differences in total mRNA or bulk protein levels for Ste12 and Tec1 in the *rpb1-CTD11* mutant compared to wild type (S5A–S5D Fig). To determine if the relative occupancy of Ste12 or Tec1 was altered at individual genes, their occupancy profiles were determined using ChIP-on-chip. Overall, the ChIP-on-chip profiles of Ste12 and Tec1 were consistent with previously reported profiles and their known relationship to Cdk8 [50,52] (S6 Fig). Although we observed alterations to Ste12 and Tec1 occupancy in the *rpb1-CTD11* mutant, these did not easily explain the increased Ty1 mRNA levels in this mutant (Fig 8A–8D). However, in the *cdk8Δ* mutant the levels of Ste12 correlated with the increased Ty1 and Ty2 mRNA levels observed, and their normalization upon truncation of the RNAPII-CTD (Fig 8A, 8C and 8E). Consistent with Cdk8’s role in targeting Ste12 for degradation [50], the *cdk8Δ* mutant had significantly increased Ste12 occupancy and increased mRNA levels at Ty1 and Ty2 elements. Upon truncation of the RNAPII-CTD, Ty1 and Ty2 mRNA levels were decreased, as were Ste12 levels at Ty1 and Ty2 promoters. Thus, in the *cdk8Δ* mutant, CTD length-dependent increases in Ste12 occupancy likely contributed to increased Ty1 and Ty2 mRNA levels.

Finally, given that Ty1 gene expression regulation is mediated by a number of different pathways, we focused on the a1-alpha2-mediated Ty1 repression and determined whether truncation of the RNAPII-CTD also resulted in increased retrotransposons expression in this biologically distinct situation [23]. Since a1-alpha2 repression is exclusive to diploid cells, we generated diploid strains homozygous for the *rpb1-CTD11* allele and observed unaltered Ty1 mRNA levels when compared to a wild type diploid strain (S7 Fig). Therefore, the repressive effect of the a1-alpha2 repressor pair was not overcome by truncation of the RNAPII-CTD, suggesting that the increased Ty1 mRNA levels observed in haploid yeast resulted from specific alterations to Ste12/Tec1-mediated regulatory pathway.

### A broader role for *TEC1* and *STE12* in the regulatory circuitry of the RNAPII-CTD

**Fig 9:**
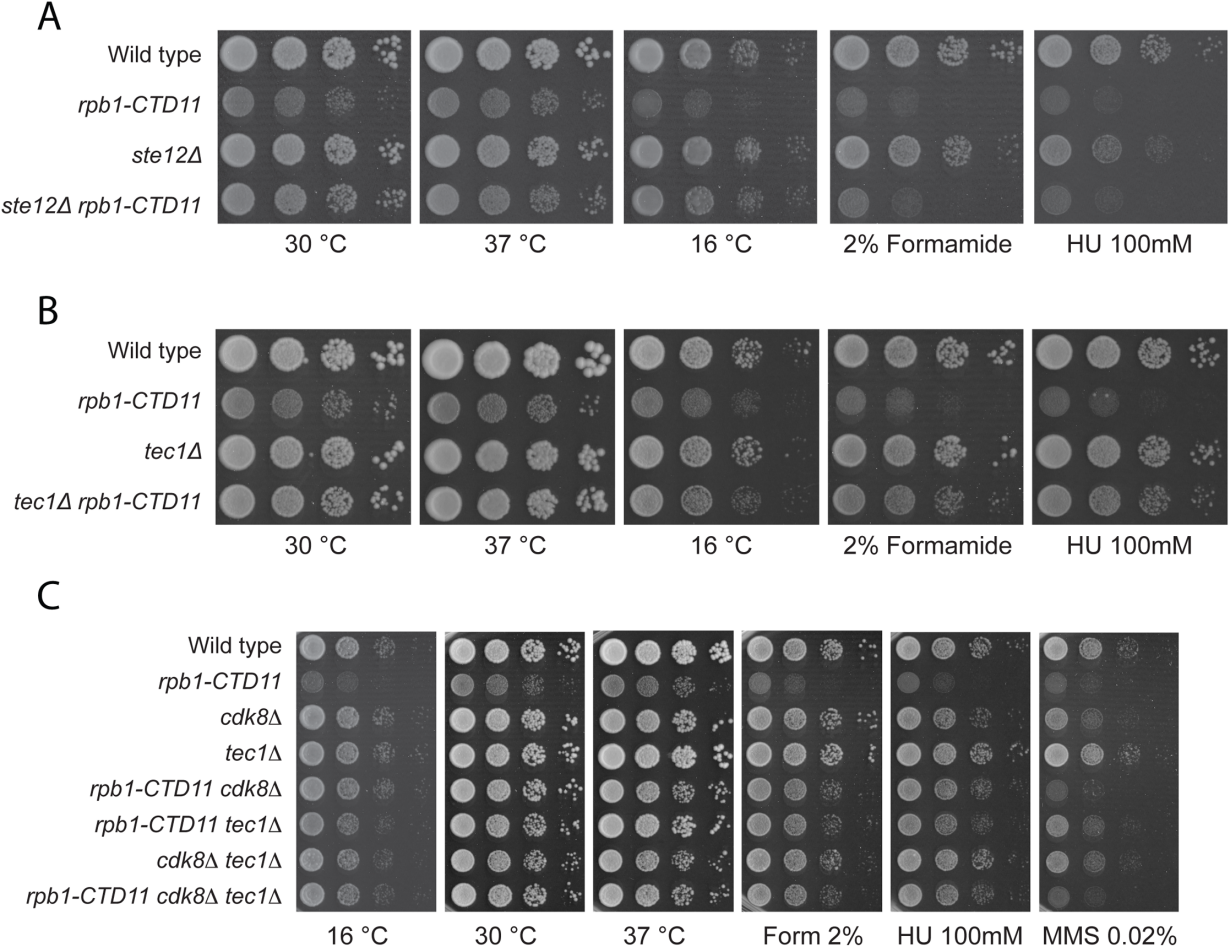
Loss of *STE12* or *TEC1* suppressed growth defects associated with *rpb1-CTD11* and the latter functioned in the same pathway as *CDK8*. (A-B) Sensitivity of the *rpb1-CTD11* mutant to growth under normal and low temperature conditions was suppressed by deletion of *STE12* or *TEC1*. Loss of *TEC1* also suppressed the growth defects of the *rpb1-CTD11* mutant upon exposure to high temperatures, formamide and hydroxyurea. Ten-fold serial dilutions of the indicated mutants were plated on YPD media at 16, 30 and 37°C or media containing the indicated concentrations of hydroxyurea or formamide. (C) Loss of *TEC1* and *CDK8* suppressed the sensitivity of the *rpb1-CTD11* mutant to growth under low and high temperatures and upon exposure to formamide and hydroxyurea. Ten-fold serial dilutions of the indicated mutants were plated and incubated on YPD media at 16, 30 and 37°C and media containing the indicated concentrations of hydroxyurea or formamide.

Truncation of the RNAPII-CTD results in both increases and decreases in gene expression under normal growth conditions [12,14]. Having established that loss of *TEC1* and *STE12* normalized the elevated expression levels of Ty1 elements caused by RNAPII-CTD truncation, we tested whether this relationship extended more broadly to other CTD length-dependent genes. Focusing on four representative protein-coding genes whose expression level is elevated in the *rpb1-CTD11* mutant, we found that further loss of *TEC1* showed a trend towards reduced mRNA levels, although the effects were small and not statistically significant (S8A-S8D Fig) [12]. As shown by RT-qPCR and sequencing of genomic DNA, loss of *TEC1* also did not normalize the reduced *RPB1* levels observed in the *rpb1-CTD11* mutant, nor did it affect the truncation status of the *rpb1-CTD11* allele (S8E Fig). Despite a small effect at representative genes, loss of *STE12* or *TEC1* robustly suppressed growth phenotypes associated with CTD truncations, suggesting a broader involvement in the cellular manifestation of truncating the RNAPII-CTD. Specifically, deletion of *STE12* or *TEC1* in the *rpb1-CTD11* background robustly normalized the slow growth phenotype of the *rpb1-CTD11* mutants when grown at 30 and 16°C (Fig 9A and 9B). In contrast to *STE12*, loss of *TEC1* also suppressed the growth defects of *rpb1-CTD11* mutants when grown at 37°C and when exposed to genotoxic agents such as hydroxyurea or formamide, indicating that the *tec1Δ* mutation was a more robust suppressor of *rpb1-CTD11* growth phenotypes than *STE12*. Overall, the suppression pattern observed for loss of *TEC1* was similar to that previously reported by loss of *CDK8* [12]. Therefore, we used genetic analysis to test whether this was achieved via independent or overlapping pathways. The strength and condition spectrum of suppression in the *rpb1-CTD11 cdk8Δ tec1Δ* triple mutant was similar to that of the *rpb1-CTD11 cdk8Δ* and *rpb1-CTD11 tec1Δ* double mutants respectively (Fig 9C). Thus, *CDK8* and *TEC1* were epistatic when suppressing RNAPII-CTD truncation phenotypes, suggesting that they functioned in the same pathway.

## Discussion

The work presented here highlights an unexpected role for the RNAPII-CTD in the regulation of retrotransposons, leading us to propose that by limiting retrotransposon gene expression, the RNAPII-CTD plays an important role in the maintenance of genomic integrity. Several lines of evidence pointed to a direct role for the RNAPII-CTD in restricting retrotransposon mobility and gene expression. First and foremost, truncation of the RNAPII-CTD unmasked this inhibitory role as it caused a significant increase in the rate of transposition of Ty1 elements. Second, higher mRNA and RNAPII occupancy levels underpinned this effect across different families of retrotransposons. Third, Cdk8 regulated the high RNAPII occupancy and mRNA expression caused by shortening the RNAPII-CTD, at least in part through promoter-mediated events. Furthermore, the close regulatory circuitry between the RNAPII-CTD, Tec1 and Cdk8 was not limited to retrotransposon expression, as loss of *TEC1* suppressed additional CTD truncation phenotypes in a manner similar to loss of *CDK8*.

Our key finding, that various aspects of RNAPII-CTD integrity were important for inhibiting retrotransposition, is consistent with an increasing appreciation of a broader involvement of RNAPII and its C-terminus in diverse aspects related to the maintenance of genome integrity. For example, yeast strains with shortened RNAPII-CTDs are sensitive to several DNA damaging drugs, including the DNA replication inhibitor hydroxyurea [15]. Interestingly, we found that these sensitivities were suppressed by loss of *TEC1*. Furthermore, strains with critically short CTDs spontaneously revert to RNAPIIs with increased CTD lengths, suggesting enhanced facility for genomic rearrangements [11]. A role for the RNAPII-CTD is also evident in the critical process of transcription coupled repair, a process which preferentially monitors the integrity of biologically relevant loci that if damaged result in RNAPII stalling, a signal for DNA repair [53]. Repair is attempted first by the nucleotide excision repair pathway, and if unsuccessful, by other repair mechanisms which first require poly-ubiquitination- and proteasome-dependent removal of RNAPII from the template [53]. The latter is dependent on the phosphorylation status of the RNAPII-CTD, which regulates the recruitment and activity of key factors involved in RNAPII ubiquitination such as the E3 ubiquitin ligase, Rsp5 [54].

We observed a 3-fold increase in Ty1 mobility in strains with truncated RNAPII-CTDs, an effect within the range but at the lower end of retrotransposon mobility spectrum [38]. The effect of the *rbp1-CTD11* mutant on Ty1 mobility was comparable to that of deleting other classical Ty1 regulators like *RTT106*. The increase in transposition, cause by altering CTD-length, was most likely a result of increased Ty1 mRNA levels due to higher levels of transcription. Consistent with this, the increased RNAPII levels at Ty elements and concomitant mRNA increases in the *rpb1-CTD11* mutant were restored to wild type levels upon loss of the transcription factors Tec1 or Ste12, or the mediator subunit Cdk8. Recapitulation of the increased expression and its dependency on Tec1 in a Ty1 promoter reporter assay provided further support for this mechanism. Furthermore, the increased levels of RNAPII at lone LTRs supported our reporter assays by revealing that the core promoter sequences were sufficient for the initial recruitment of RNAPII with shortened CTDs. However, our analysis of these sites also suggested a nuanced mechanism of Ty1 transcription activation. Specifically, lone LTR genomic loci lack functional Ste12/Tec1 binding sites which tend to be located downstream of the ATG translation start codon. Reconciling this with a clear requirement for Ste12 and Tec1 in mediating the increased expression levels at Ty1 retrotransposons caused by shortening the RNAPII-CTD, suggested that Ste12 and Tec1 functioned to enhance transcription complex assembly on the core promoter sequence. In support of this model, *cdk8Δ* mutants, which had increased Ste12 occupancy at the promoter also showed increased RNAPII levels at the 5’ end of Ty1 and Ty2 elements, indicative of higher rates of initiation. Thus, these data point to a multi layer approach to transcriptional control at Ty1 promoters, where sequences upstream of the ATG start codon were sufficient for RNAPII recruitment, but additional regulatory layers down-stream functioned to increase rates of transcription initiation. These observations are consistent with previous reports that indicated that the full integrity of the Ty1 promoter was important for full activation [24]. Finally, our results suggest that this model also extends to Ty2 elements, even though differences in their regulation have been reporter. Primarily Ty1 elements depend on *TEC1* for their expression while Ty2 elements do not [28].

**Fig 10:**
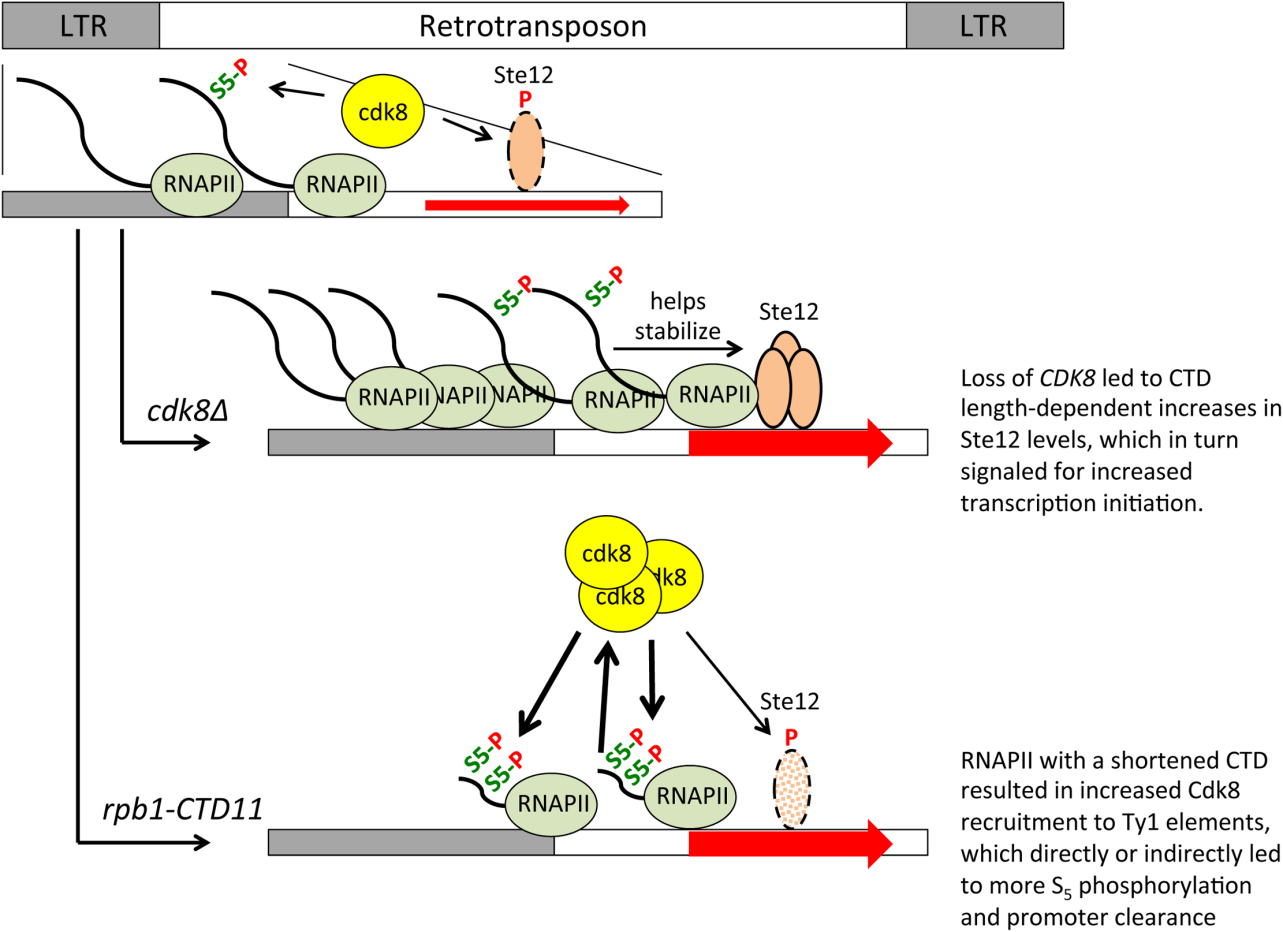
A role for the RNAPII-CTD and Cdk8 in Ty1 gene expression regulation. Cdk8 normally functions to target Ste12 for degradation, thus loss of *CDK8* stabilized Ste12 at Ty1 promoters resulting in increased transcription initiation. Stability of Ste12 at Ty1 promoters was dependent on full length RNAPII-CTD, thus in the *rpb1-CTD11 cdk8Δ* double mutant Ste12 levels were normalized leading to wild type levels of transcription initiation. Truncation of the RNAPII-CTD alone resulted in increased Cdk8 recruitment to Ty1 elements and increased S_5_ phosphorylation levels, a key mark for promoter clearance. The increased S_5_ phosphorylation levels in the *rpb1-CTD11* mutant were direct or indirectly dependent on Cdk8, thus in the *rpb1-CTD11 cdk8Δ* double mutant S_5_ phosphorylation levels were normalized resulting in decreased levels of promoter clearance.

The effect of the RNAPII-CTD on Ty1 gene expression was reminiscent of the role of the RNAPII-CTD on a subset of Rpn4-regulated genes. Specifically, under normal growth conditions Rpn4-regulated genes [12] and retrotransposons had increased RNAPII and mRNA levels in the *rpb1-CTD11* mutant which were dependent on *CDK8*, and were mediated by alterations to transcription initiation. However, despite the similarities, distinct roles of Cdk8 suggested different transcriptional regulatory processes. Specifically, while Cdk8 was normally present at Rpn4-regulated genes, its loss did not change their expression level. In contrast, at Ty1 elements, Cdk8 levels increased upon truncation of the RNAPII-CTD and Cdk8 alone played a role in their regulation as evidenced by increased RNAPII and mRNA levels in the *CDK8* deletion mutant. The increased Ty1 and Ty2 mRNA levels observed in the *cdk8Δ* mutant compared to the wild type, likely resulted from its function as a negative regulator of Ste12 protein levels [50]. As such, Cdk8 normally functioned to repress Ty1 mRNA levels and its loss led to increased Ste12 levels and transcription initiation (Fig 10). Importantly, the effect of Cdk8 on Ste12 was modulated by RNAPII-CTD length, resulting in decreased Ste12 and Ty1 and Ty2 mRNA levels in the *rpb1-CTD11 cdk8Δ* double. A different mechanism of Ty1 gene expression regulation was elicited when the RNAII-CTD was truncated. Although truncation of the RNAPII-CTD also led to increased Ty1 mRNA levels, these were not a result of increased Ste12 occupancy. In fact, we observed lower than normal Ste12 levels at Ty1 retrotransposons in the *rpb1-CTD11* mutant, an effect likely mediated by the observed increase in Cdk8 recruitment to Ty1 and Ty2 elements. Instead, the increased Ty1 mRNA levels in the *rpb1-CTD11* mutant correlated with increased S_5_ phosphorylation levels, a post initiation event associated with promoter clearance. Thus, it is likely that truncating the RNAPII-CTD elevated the rates of RNAPII promoter clearance, resulting in increased transcription, an effect that was in turn dependent on *CDK8*. Highlighting two distinct roles for Cdk8 in the regulation of Ty1 gene expression, it also functioned as an activator by stimulating RNAPII-CTD S_5_ phosphorylation and promoter release. The latter is consistent with its reported role for some protein-coding genes in mammals [55,56]. This model would also be consistent with the reciprocal suppression between Cdk8 and the RNAPII-CTD we observed at Ty1 and Ty2 elements.

It is unclear to what extent the RNAPII-CTD length-dependent transcriptional regulatory pathways are linked. Integration of retrotransposons at transcription regulatory sites can alter the transcription of adjacent genes and thus alterations to Ty1 gene expression regulation and transpositions could underlie some of the observed transcriptional defects observed at other genes [12,20]. However, this effect is limited to genes near retrotransposons and we observed no correlation between being in the vicinity of a retrotransposon and having altered mRNA levels in the *rbp1-CTD11* mutant. Suggestive of a different type of connection, we noted that truncation of the RNAPII-CTD resulted in *STE12*-dependent increases in Ty1 mRNA levels and a concomitant decreased expression of protein-coding genes primarily regulated by Ste12 [12]. Given that bulk Ste12 levels were unaltered in the *rbp1-CTD11* mutant, one possibility is that the increased transcriptional output at Ty1 elements reduced the cellular pool of Ste12 protein necessary to drive the expression of other genes. Careful examination of the Ste12 ChIP-on-chip profiles did not support this hypothesis indicating that more work beyond the scope of this investigation will be necessary to illuminate the degree of network connectivity between the distinct transcriptional programs found in the *rpb1-CTD11* mutant strains, and their detailed mechanistic underpinning. Nonetheless, finding that *TEC1* acted as a gene with classical SRB-like phenotypes in that it could suppress the temperature, HU, and formamide sensitivity of the *rpb1-CTD11* mutant, suggested that it played fundamental roles in RNAPII-CTD biology. In addition, our results indicate that *CDK8*, the RNAPII-CTD, *TEC1*, and to a lesser extent *STE12*, worked together in a broad network aimed at maintaining genome integrity, which in part involved limiting genome instability caused by Ty1 mobility.

## Materials and Methods

### Yeast strains

Strains are listed in Table 4. Complete or partial gene deletions were achieved via the one-step gene replacement method [57]. CTD truncations were generated previously by addition of a TAG stop codon followed by a NAT, kanamycin or hygromycin resistance marker at the endogenous *RPB1* locus [12]. All strains containing the *rpb1-CTD11* allele were confirmed by sequencing. All double mutant strains were generated via mating and tetrad dissection. For *STE12* deletion mutants, strains were complemented with pRS316 [STE12] prior to mating. The pRS316 [STE12] plasmid was a gift from Dr. Ivan Sadowski and the pJC573—Ty1his3AI*[Δ1]* plasmid was a gift from Dr. Joan Curcio. The *rpb1-S2A* and *rpb1-S7A* mutants were obtained from Dr. Damien Herman. Plasmids are listed in Table 5.

**Table 4:** Strains used in this study.

Number	Genotype	Background	Reference
MKY1507	Matα his3Δ1 leu2Δ0 ura3Δ0	BY4742	Aristizabal *et al* 2013
MKY1508	Matα rpb1-CTD11-nat his3Δ1 leu2Δ0 ura3Δ0	BY4742	Aristizabal *et al* 2013
MKY1513	Matα rpb1-CTD11-nat cdk8::kan his3Δ1 leu2Δ0 ura3Δ0	BY4742	Aristizabal *et al* 2013
MKY1515	Matα cdk8::kan his3Δ1 leu2Δ0 ura3Δ0	BY4742	van de Peppel *et al* 2005
MKY1710	Matα tec1::kan his3Δ1 leu2Δ0 ura3Δ0	BY4742	This study
MKY1711	Matα ste12::kan his3Δ1 leu2Δ0 ura3Δ0	BY4742	This study
MKY1712	Matα rpb1-CTD11-nat tec1::kan his3Δ1 leu2Δ0 ura3Δ0	BY4742	This study
MKY1713	Matα rpb1-CTD11-nat ste12::kan his3Δ1 leu2Δ0 ura3Δ0	BY4742	This study
MKY1714	Matα tec1::hygro his3Δ1 leu2Δ0 ura3Δ0	BY4742	This study
MKY1715	Matα rpb1-CTD11-nat tec1::hygro his3Δ1 leu2Δ0 ura3Δ0	BY4742	This study
MKY1716	Matα cdk8::kan tec1::hygro his3Δ1 leu2Δ0 ura3Δ0	BY4742	This study
MKY1717	Matα rpb1-CTD11-nat cdk8::kan tec1::hygro his3Δ1 leu2Δ0 ura3Δ0	BY4742	This study
MKY1718	MATa/α his3Δ1/his3Δ1 leu2Δ0/leu2Δ0 LYS2/lys2Δ0	BY4743	This study
MKY1719	MATa/α rpb1-CTDWT-nat rpb1-CTDWT-kan his3Δ1/his3Δ1 leu2Δ0/leu2Δ0 LYS2/lys2Δ0	BY4743	This study
MKY1721	MATa/α rpb1-CTD11-nat rpb1-CTD11-kan his3Δ1/his3Δ1 leu2Δ0/leu2Δ0 LYS2/lys2Δ0	BY4743	This study
MKY1790	Matα STE12-Flag-hygro his3Δ1 leu2Δ0 ura3Δ0	BY4742	This study
MKY1791	Matα STE12-Flag-hygro rpb1-CTD11-nat his3Δ1 leu2Δ0 ura3Δ0	BY4742	This study
MKY1792	Matα STE12-Flag-hygro cdk8::kan his3Δ1 leu2Δ0 ura3Δ0	BY4742	This study
MKY1793	Matα STE12-Flag-hygro rpb1-CTD11-nat cdk8::kan his3Δ1 leu2Δ0 ura3Δ0	BY4742	This study
MKY1794	Matα TEC1-Flag-hygro his3Δ1 leu2Δ0 ura3Δ0	BY4742	This study
MKY1795	Matα TEC1-Flag-hygro rpb1-CTD11-nat his3Δ1 leu2Δ0 ura3Δ0	BY4742	This study
MKY1796	Matα TEC1-Flag-hygro cdk8::kan his3Δ1 leu2Δ0 ura3Δ0	BY4742	This study
MKY1797	Matα TEC1-Flag-hygro rpb1-CTD11-nat cdk8::kan his3Δ1 leu2Δ0 ura3Δ0	BY4742	This study
MKY1516	Matα CDK8-Flag::kan his3Δ 1 leu2Δ 0 ura3Δ 0	BY4742	Aristizabal *et al* 2013
MKY1561	Matα CDK8-Flag-kan rpb1-CTD11-nat his3Δ1 leu2Δ0 ura3Δ0	BY4742	Aristizabal *et al* 2013
MKY1562	Matα SUA7-Flag-kan his3Δ1 leu2Δ0 ura3Δ0	BY4742	Aristizabal *et al* 2013
MKY1563	Matα SUA7-Flag-kan rpb1-CTD11-nat his3Δ1 leu2Δ0 ura3Δ0	BY4742	Aristizabal *et al* 2013
MKY1564	Matα CET1-Flag-kan his3Δ1 leu2Δ0 ura3Δ0	BY4742	Aristizabal *et al* 2013
MKY1565	Matα CET1-Flag-kan rpb1-CTD11-nat his3Δ1 leu2Δ0 ura3Δ0	BY4742	Aristizabal *et al* 2013
MKY1566	Matα ELF1-Flag-kan his3Δ1 leu2Δ0 ura3Δ0	BY4742	Aristizabal *et al* 2013
MKY1567	Matα ELF1-Flag-kan rpb1-CTD11-nat his3Δ1 leu2Δ0 ura3Δ0	BY4742	Aristizabal *et al* 2013
MKY1798	MATa ura3 leu2 his3 met15	BY4741	Cassart *et al* 2012
MKY1799	MATa rpb1-S2A-kan ura3 leu2 his3 met15	BY4741	Cassart *et al* 2012
MKY1800	MATa rpb1-S7A-kan ura3 leu2 his3 met15	BY4741	Cassart *et al* 2012

**Table 5:** Plasmids used in this study.

Plasmid number	Relevant Genotype	Backbone	Source
pMK626	YJRWTy1-2 promoter	pGL669-z	This study
pMK627	YMLWTy1-2 promoter	pGL669-z	This study
pMK628	STE12	pRS316	Dr. Ivan Sadowski
pMK629	YJRWTy1-2 promoter Tec1 binding site deletions	pGL669-z	This study
pMK630	YMLWTy1-2 promoter Tec1 binding site deletions	pGL669-z	This study
pMK631	pJC573—Ty1*his3AI[Δ1]*	pRS406	Bryk *et al* 2001
pMK649	YDRWTy2-2 promoter	pGL669-z	This study
pMK650	YLRWTy2-1 promoter	pGL669-z	This study

### Genome-wide ChIP-on-chip

Rpb3 and transcription associated factor ChIP-on-chip data used were generated previously [12]. Complete datasets can be found in array-express, code E-MTAB-1341, E-MTAB-1379, and E-MTAB-3906. Briefly, overnight cultures were diluted to 0.15 OD600 and grown to 0.5–0.6 OD600 units. Cross-linking was done with 1% formaldehyde for 20 min. Chromatin was prepared as described previously [58]. Five μl of anti-Rpb3 (Neoclone), or 4.2 μl of anti-FLAG (Sigma) were coupled to 60 μl of protein A Dynabeads (Invitrogen). DNA was amplified using a double T7 RNA polymerase method, biotin labeled, and hybridized to Affymetrix 1.0R *S*. *cerevisiae* microarrays. Rpb3 samples were normalized to input and flag tagged samples were normalized to a mock control using the rMAT software [59]. Relative occupancy scores were calculated for all probes using a 300 bp sliding window. Experiments were carried out in duplicate; quantile normalized and averaged data were used for calculating average enrichment scores. To obtain average RNAPII scores at retrotransposons and LTRs, we averaged probes whose start sites fell within the feature start and end positions. For the box plots, the middle line represents the median and the hinges represent the first and third quartile. To obtain average Ste12 and Tec1 occupancy scores, all probes whose start sites fell within the first 1500 bp of the retrotransposon were averaged, as such they included the 5’ LTR and about 1000 bp downstream of the start codon.

### Genome-wide ChIP-on-chip of CTD Phospho-isoforms

Cells were grown and cross-linked as described above. All buffers contained protease (roche) and phosphatase inhibitors (10 mM NaPPi, 5 mM EGTA, 5mM EDTA, 0.1 mM Sodium orthovanadate, 5 mM NaF). Chromatin was prepared as described previously [58] and incubated overnight with 50 μl of anti-phospho-serine 2 (3E10) or anti-phospho-serine 5 (3E8) antibodies [8]. Twenty ul of protein G Sepharose (GE Healthcare) was added and incubated for 1.5 hours at 4°C. Beads were washed 3 times with FA lysis buffer with protease and phosphatase inhibitors (50 mM HEPES-KOH (pH 7.5), 1 mM EDTA, 1% Triton X-100, 0.1% SDS), 3 times with FA lysis buffer plus NaCl (500mM), one times with ChIP wash buffer (10 mM Tris-HCL (pH 8.0), 0.25 M LiCl, 1mM EDTA, 0.5% NP-40, 0.5% sodium deoxycholate) followed by TE. Immunoprecipitated and purified DNA was amplified, labeled, and hybridized as described above. Phospho-Serine 2 and 5 profiles were normalized to an input or corresponding Rpb3 profiles, and processed as described above [59].

### Reporter assays

Reporter plasmids were generated by cloning ~1300 bp of the desired promoter region into the Sal1 and BamH1 sites of pLG669-Z [60]. Specifically, for YJRWTy1-2 and YMLWTy1-2 1321bp were cloned, starting at 517bp upstream of the ORF start and ending 804 bp downstream. For YLRWTy2-1 and YDRWTy2-2 1304 bp were cloned, starting 500 bp upstream of the ORF start and ending 804 bp downstream. For the Ty1 genes, the cloned sequences were selected such that they included previously reported Tec1 and Ste12 binding regions [52]. Tec1 binding sequences were deleted using nested PCR-based methods and cloned into pLG669-Z using the Sal1 and BamH1 sites. A complete list of plasmids can be found in Table 5. Reporter plasmids were transformed into the indicated mutants. Whole cell extracts were generated and clarified as described previously [61]. β-galactosidase activity was normalized to total protein levels determined using the Bradford assay. Measurements were obtained from three independent cultures and error bars represent standard deviations.

### Growth assays

Overnight cultures grown on YPD were diluted to 0.5 OD600, 10-fold serially diluted and spotted onto YPD plates with or without the indicated amounts of hydroxyurea (Sigma) or formamide (Sigma). Plates were incubated at the indicated temperatures for 2–4 days.

### Reverse Transcriptase PCR (RT-PCR)

RNA was extracted and purified using the Qiagen RNeasy Mini Kit. cDNA was generated using the Qiagen QuantiTect Reverse Transcription Kit. cDNA was analyzed using a Rotor-Gene 600 (Corbett Research) and PerfeCTa SYBR Green FastMix (Quanta Biosciences). Samples were analyzed in triplicate from three independent RNA preparations and the protein-coding gene *TUB1* was used as a control given that its expression is not altered upon truncation of the RNAPII-CTD [62]. For measuring Ty1 mRNA levels 6 pg/μl of cDNA were used in a 15 μl PCR reaction. For measuring Ty2 mRNA levels 60 pg/μl of cDNA were used. Retrotransposon specific primers were designed, such that the targeted region was unique to all members of a single retrotransposon family. Primer specificity was evaluated by melt curve analysis of the PCR products. A complete list of primers used in this study can be found in Table 6. Error bars represent standard deviations.

**Table 6:** Primers used in this study.

Primer name	Forward Sequence	Reverse Sequence	Source
Ty1	CCAGTTTGGGTGGTATTGGT	TTCTTCGATCTCGGAGGTTC	This study
Ty2	TGCCAACATGGGTAAAACAA	GGCCAATCTGTCGCTAACAT	This study
*TUB1*	TCTTGGTGGTGGTACTGGTT	TGGATTTCTTACCGTATTCAGCG	Lu and Kobor 2014
*STE12*	CGATGTTCCCATACATGCAA	CAGACTTGCCCACAGATTGTT	This study
*TEC1*	GGCCTCTTGAACAACTTTCC	CGGAATTGGAATTGGAACTG	This study
*YCR061W*	GGCCACAGACCGATGTAGTT	TGTTAGCAGACGGAAGAAGGA	This study
*YML116W*	CACCGGTTCACGAGACATAC	ACCCATACCGAGACACAAGG	This study
*YIR034C*	CTGCCGGGCCTAAATTATCT	ACGAGCGCAATGTCTATCG	This study
*YKL145W*	GGTGAAGGTGCTCGTATGGT	GGGTCAAACCCGTCTAACTG	This study
*RPB1*	CAGGCATTCGATTGGGTATT	GGAAGCAACACCAGCAAAAT	This study

### Ty1 cDNA-mediated mobility assay

This assay tracks the mobility of a genome encoded Ty1 element [38,39]. The element has a *HIS3* coding sequence inserted in the opposite orientation compared to the Ty1 element. The *HIS3* gene is rendered nonfunctional by the insertion of an artificial intron in the same orientation as the Ty1 element, such that it is only spliced when transcribed from the Ty1 element. During the Ty1 transposition cycle, the Ty1 element is transcribed, the intron is spliced and the mature RNA is used for the synthesis of cDNA, which is then integrated into a new genomic location. Newly integrated Ty1 elements contain an undisrupted *HIS3* open reading frame and can confer a HIS+ phenotype. Briefly, wild type and *rpb1-CTD11* mutants were transformed with Pac1 digested pJR573 DNA. Transformants were selected in SC-URA media and all subsequent growth procedures were done in this media. Overnight cultures for 12 independent colonies for each strain were started. The next morning, cultures were diluted to OD600 0.3 and incubated at 20°C for 24 hours. Following, an aliquot was plated onto SC-URA and SC-HIS-URA to count the total number of cells, and cells with retrotransposition events, respectively. Plates were grown for 2–3 days until colonies were visible and counted. Results were analyzed using the Fluctuation AnaLysis CalculatOR (FALCOR) web tool using the MSS Maximum Likelihood Method to calculate mutation rates. [63]. Error bars represent 95% confidence intervals as calculated by the FALCOR web tool. http://www.mitochondria.org/protocols/FALCOR.html

## Supporting Information

S1 Fig Truncation of the RNAPII-CTD resulted in increased RNAPII levels at Ty3 elements. Chromosome plots of relative RNAPII occupancy at both Ty3 retrotransposons in the *S*. *cerevisiae* genome. Increased RNAPII levels were observed at the 5’ and 3’ end of these elements in the *rpb1-CTD11* mutant compared to wild type. Labeled boxes indicate the retrotransposon.
(PDF)

S2 Fig Lone LTRs derived from Ty1 and Ty2 elements showed increased RNAPII levels in the *rpb1-CTD11* mutant when compared to wild type. Box plot showing differences in average MAT RNAPII occupancy scores between the wild type and the *rpb1-CTD11* mutant strain at all, Ty1-, or Ty2-derived lone LTRs.
(PDF)

S3 Fig Profiles of RNAPII-CTD phospho-S_2_ and S_5_ occupancy under wild type conditions were consistent with previously reported profiles. (A) CHROMATRA plots [64] of RNAPII-CTD phosphor-S_2_ profiles normalized to input or RNAPII levels revealed strong enrichment toward the 3’ end of genes. Genes in the *S*. *cerevisiae* genome are aligned by their transcriptional start side (TSS), grouped into transcriptional frequency categories [65], and sorted by gene length. (B) CHROMATRA plots for RNAPII-CTD phosphor-S_5_ profiles normalized to input (left) or RNAPII levels (right) revealed strong occupancy at the 5’ end of genes.
(PDF)

S4 Fig Similar trends where observed when RNAPII-CTD S_5_ phosphorylation ChIP-on-chip profiles were normalized to Rpb3 levels. (A) Box plot showing differences in average RNAPII-CTD S_5_ phosphorylation occupancy scores in the *rpb1-CTD11*, *cdk8Δ* and *rpb1-CTD11 cdk8Δ* mutant at Ty1 retrotransposons. (B) Average gene profiles of RNAPII-CTD phospho-S_5_ occupancy at Ty1 retrotransposons revealed elevated levels in the *rpb1-CTD11* mutant which were normalized upon loss of *CDK8*.
(PDF)

S5 Fig Truncating the RNAPII-CTD did not alter the mRNA or protein levels of Ste12 or Tec1. RT-qPCR analysis of Ste12 (A) or Tec1 (B) mRNA levels in the *rpb1-CTD11* mutant compared to wild type. Immunoblots of whole cell extracts with flag antibodies to detect Ste12 (C) or Tec1 (D) protein levels. Tubulin was used as a loading control.
(PDF)

S6 Fig Ste12 and Tec1 ChIP-on-chip profiles were consistent with previously reported profiles and identified known Ste12 and Tec1 regulated genes. (A) Venn diagram highlighting a significant overlap between genes we identified to be significantly bound by Ste12 or Tec1, an effect consistent with previous reports [52]. Venn diagrams displaying the overlap between genes identified as significantly bound by Ste12 (B) or Tec1 (C) in our profiles and those reportedly bound by these factors in the YEASTRACT database (http://www.yeastract.com/). Average gene profile of gene identified by our data to be significantly bound by Ste12 (D) or Tec1 (E) in the wild type, *rpb1-CTD11*, *cdk8Δ* and *rpb1-CTD11 cdk8Δ* double mutant. Box plots showing significantly increased Ste12 (F) or Tec1 (G) binding at Ste12 or Tec1 regulated genes respectively in the *cdk8Δ* mutant compared to wild type.
(PDF)

S7 Fig Repression by the a1-alpha2 repressor pair was not overcome by truncation of the RNAPII-CTD. RT-qPCR analysis of the indicated diploid strains.
(PDF)

S8 Fig Loss of *TEC1* suppressed additional gene expression alterations observed in the *rpb1-CTD11* mutant. (A-D) RT-qPCR analysis of YKL145W, YIR034C, YML116W, and YCR061W mRNA levels in wild type, *rpb1-CTD11*, *tec1Δ* and *rpb1-CTD11 tec1Δ* mutants. (E) Decreased levels of *RPB1* in the *rpb1-CTD11* mutant were not normalized upon loss of *TEC1*. The *RPB1* RT-qPCR primers were designed such that they bound a region upstream of the sequence coding for the CTD.
(PDF)
