# Supplementary figures and images for: The RNAPII-CTD Maintains Genome Integrity through Inhibition of Retrotransposon Gene Expression and Transposition
Source: PLoS Genet. 2015 Oct 23;11(10):e1005608. doi: 10.1371/journal.pgen.1005608 (PMC4619828; doi:10.1371/journal.pgen.1005608)

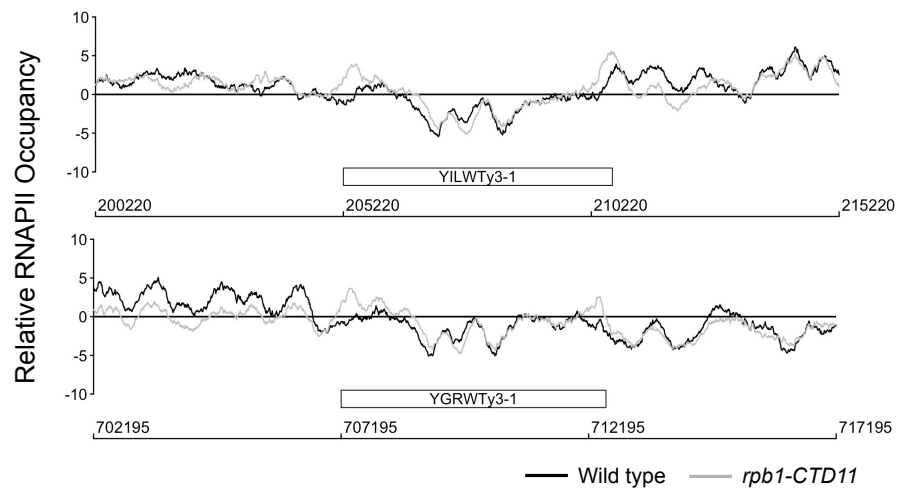

Supplement: S1 Fig — Chromosome plots of relative RNAPII occupancy at both Ty3 retrotransposons in the S. cerevisiae genome. Increased RNAPII levels were observed at the 5’ and 3’ end of these elements in the rpb1-CTD11 mutant compared to wild type. Labeled boxes indicate the retrotransposon. (PDF) [file pgen.1005608.s001.pdf]

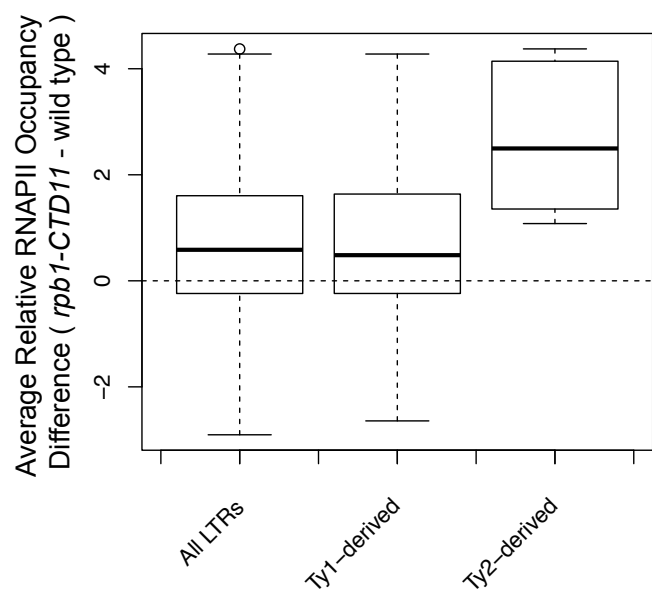

Supplement: S2 Fig — Box plot showing differences in average MAT RNAPII occupancy scores between the wild type and the rpb1-CTD11 mutant strain at all, Ty1-, or Ty2-derived lone LTRs. (PDF) [file pgen.1005608.s002.pdf]

Phospho-S2 (3E10)

A

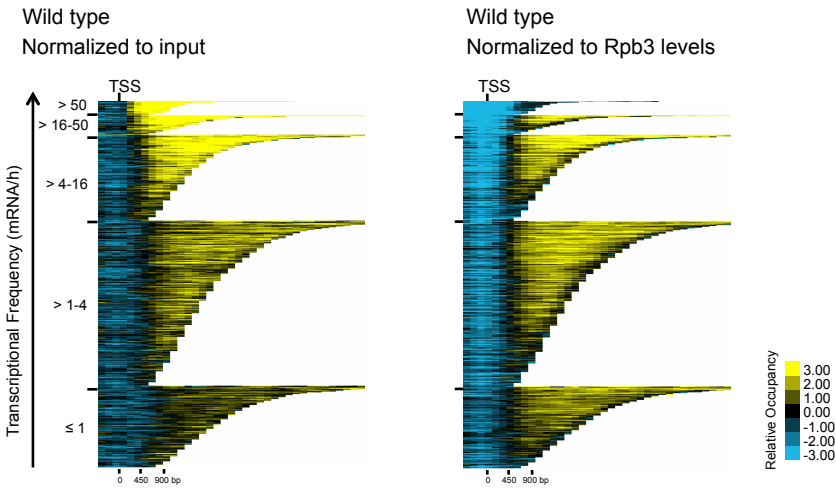

Phospho-S5 (3E8)

B

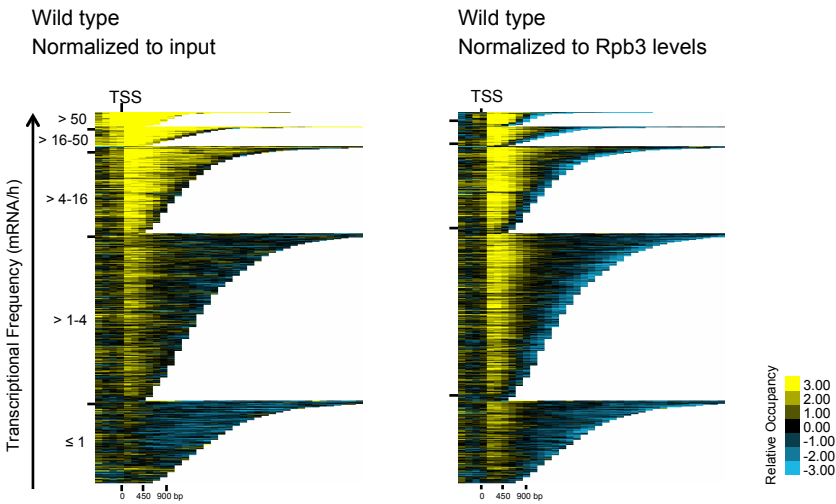

Supplement: S3 Fig — (A) CHROMATRA plots [64] of RNAPII-CTD phosphor-S2 profiles normalized to input or RNAPII levels revealed strong enrichment toward the 3’ end of genes. Genes in the S. cerevisiae genome are aligned by their transcriptional start side (TSS), grouped into transcriptional frequency categories [65], and sorted by gene length. (B) CHROMATRA plots for RNAPII-CTD phosphor-S5 profiles normalized to input (left) or RNAPII levels (right) revealed strong occupancy at the 5’ end of genes. (PDF) [file pgen.1005608.s003.pdf]

A

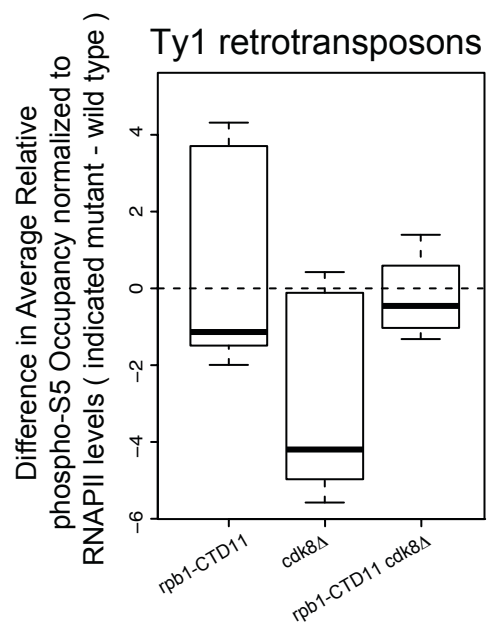

B

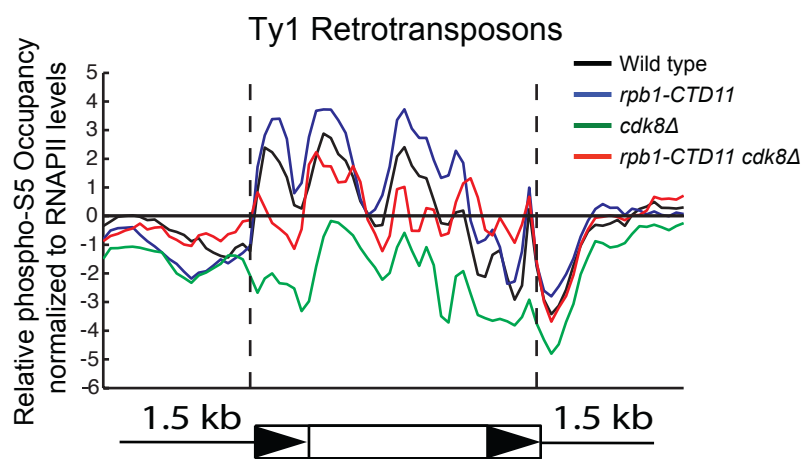

Supplement: S4 Fig — (A) Box plot showing differences in average RNAPII-CTD S5 phosphorylation occupancy scores in the rpb1-CTD11, cdk8Δ and rpb1-CTD11 cdk8Δ mutant at Ty1 retrotransposons. (B) Average gene profiles of RNAPII-CTD phospho-S5 occupancy at Ty1 retrotransposons revealed elevated levels in the rpb1-CTD11 mutant which were normalized upon loss of CDK8. (PDF) [file pgen.1005608.s004.pdf]

A

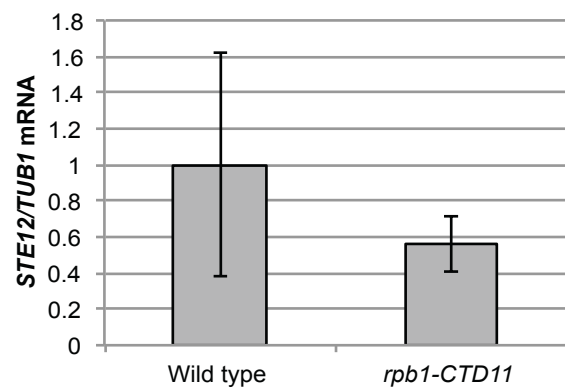

B

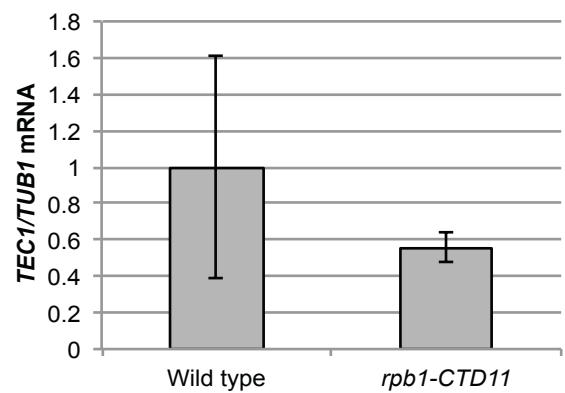

C

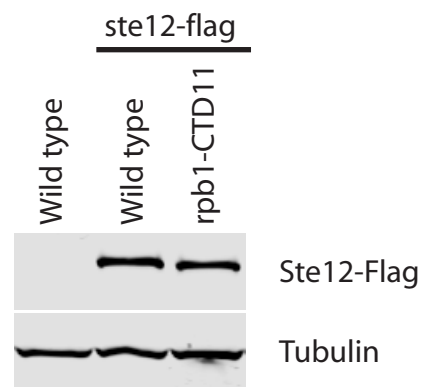

D

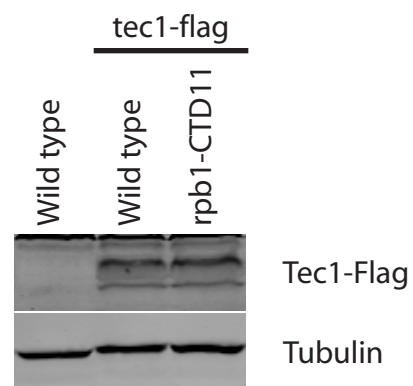

Supplement: S5 Fig — RT-qPCR analysis of Ste12 (A) or Tec1 (B) mRNA levels in the rpb1-CTD11 mutant compared to wild type. Immunoblots of whole cell extracts with flag antibodies to detect Ste12 (C) or Tec1 (D) protein levels. Tubulin was used as a loading control. (PDF) [file pgen.1005608.s005.pdf]

Ty1 mRNA Levels

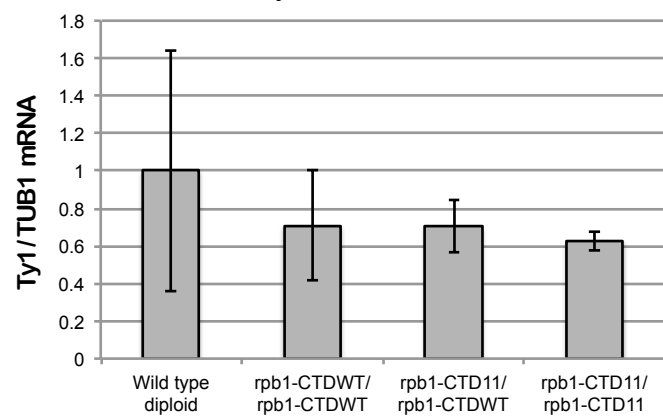

Supplement: S7 Fig — RT-qPCR analysis of the indicated diploid strains. (PDF) [file pgen.1005608.s007.pdf]

A

YCR061W mRNA level

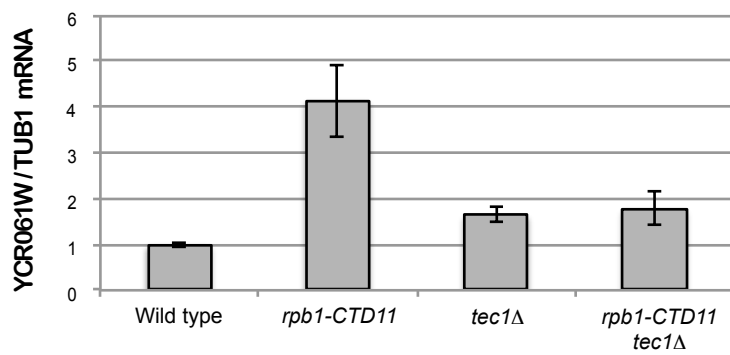

B

YML116W mRNA level

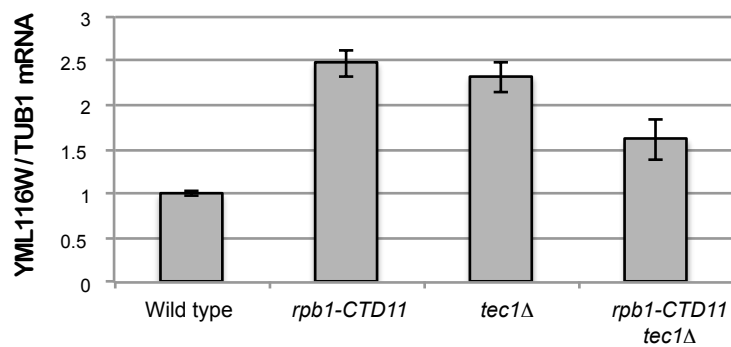

C

YIR034C mRNA level

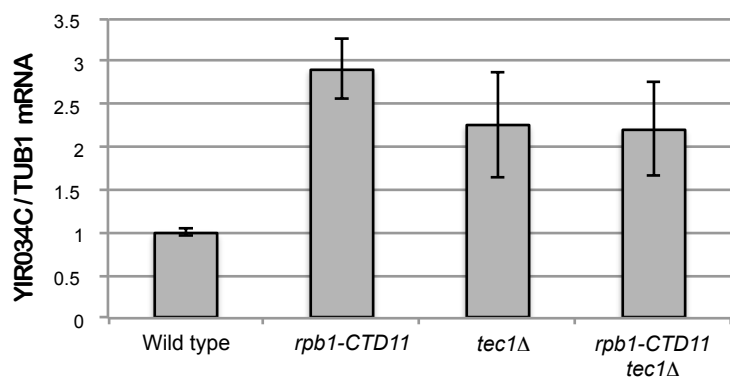

D

YKL145W mRNA level

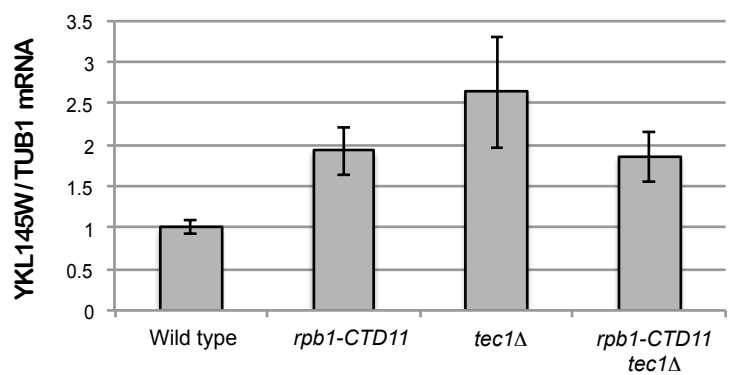

E

RPB1 mRNA level

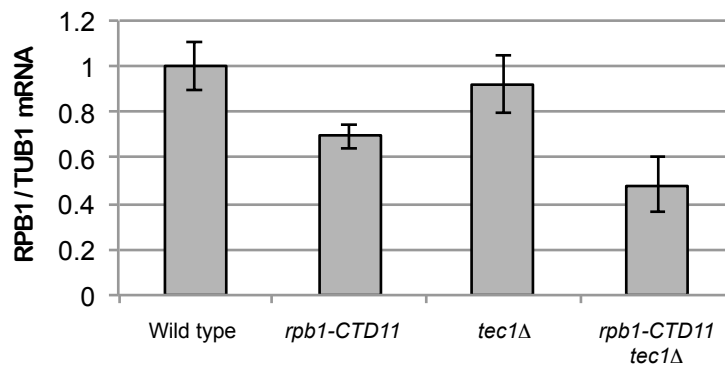

Supplement: S8 Fig — (A-D) RT-qPCR analysis of YKL145W, YIR034C, YML116W, and YCR061W mRNA levels in wild type, rpb1-CTD11, tec1Δ and rpb1-CTD11 tec1Δ mutants. (E) Decreased levels of RPB1 in the rpb1-CTD11 mutant were not normalized upon loss of TEC1. The RPB1 RT-qPCR primers were designed such that they bound a region upstream of the sequence coding for the CTD. (PDF) [file pgen.1005608.s008.pdf]
