# Supplementary material for: The RNAPII-CTD Maintains Genome Integrity through Inhibition of Retrotransposon Gene Expression and Transposition
Source: PLoS Genet. 2015 Oct 23;11(10):e1005608. doi: 10.1371/journal.pgen.1005608 (PMC4619828; doi:10.1371/journal.pgen.1005608)
Supplement: S6 Fig — (A) Venn diagram highlighting a significant overlap between genes we identified to be significantly bound by Ste12 or Tec1, an effect consistent with previous reports [52]. Venn diagrams displaying the overlap between genes identified as significantly bound by Ste12 (B) or Tec1 (C) in our profiles and those reportedly bound by these factors in the YEASTRACT database (http://www.yeastract.com/). Average gene profile of gene identified by our data to be significantly bound by Ste12 (D) or Tec1 (E) in the wild type, rpb1-CTD11, cdk8Δ and rpb1-CTD11 cdk8Δ double mutant. Box plots showing significantly increased Ste12 (F) or Tec1 (G) binding at Ste12 or Tec1 regulated genes respectively in the cdk8Δ mutant compared to wild type. (PDF) [file pgen.1005608.s006.pdf]

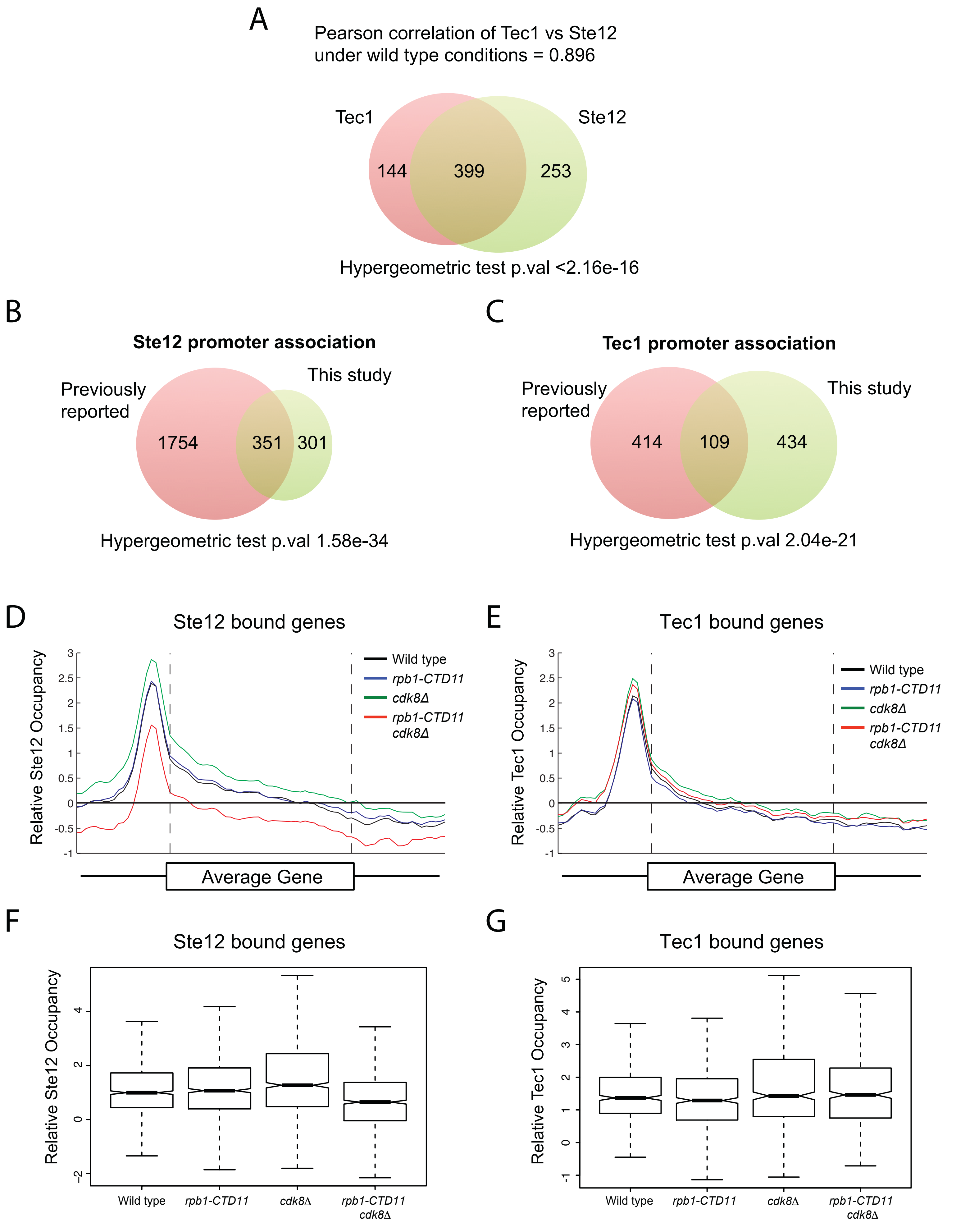

A
Pearson correlation of Tec1 vs Ste12
under wild type conditions = 0.896
Tec1
Ste12
144
399
253
Hypergeometric test p.val <2.16e-16
B
Ste12 promoter association
Previously reported
This study
1754
351
301
Hypergeometric test p.val 1.58e-34
C
Tec1 promoter association
Previously reported
This study
414
109
434
Hypergeometric test p.val 2.04e-21
D
Ste12 bound genes
Relative Ste12 Occupancy
Wild type
rpb1-CTD11
cdk8Δ
rpb1-CTD11 cdk8Δ
Average Gene
E
Tec1 bound genes
Relative Tec1 Occupancy
Wild type
rpb1-CTD11
cdk8Δ
rpb1-CTD11 cdk8Δ
Average Gene
F
Ste12 bound genes
Relative Ste12 Occupancy
Wild type
rpb1-CTD11
cdk8Δ
rpb1-CTD11 cdk8Δ
G
Tec1 bound genes
Relative Tec1 Occupancy
Wild type
rpb1-CTD11
cdk8Δ
rpb1-CTD11 cdk8Δ
